# Trends in educational inequalities in all-course mortality and deaths of despair in Swedish youths 1990–2018

**DOI:** 10.1016/j.ssmph.2025.101748

**Published:** 2025-01-04

**Authors:** Björn Högberg, Simone Scarpa, Solveig Petersen

**Affiliations:** a Department of Social Work, Umeå University, Sweden; b Centre for Demographic and Ageing Research, Umeå University, Sweden; c Department of Epidemiology and Global Health, Umeå University, Sweden

**Keywords:** Adolescents, Young adults, Achievement, Performance, Trends, Disparities

## Abstract

**Bakground:**

Growing educational disparities in mortality due to suicide, drug overdose, or alcohol-related liver disease – or “deaths of despair” (DoD) – have received increased attention in research and public debate. However, no study has investigated educational differences in despair-related mortality outside of North America. Thus, the aim this study was to investigate changes in the association between academic achievement in compulsory school and subsequent all-cause mortality (ACM) and DoD between 1990 and 2018 in Swedish youths.

**Data and methods:**

Register data on all students graduating from compulsory school in Sweden between 1990 and 2010 were used (N = 2 252 938). Students were followed for a maximum of 8 years using discrete time proportional hazard models. Academic achievement was measured by grades at the end of compulsory school.

**Results:**

ACM declined for medium/high achieving but not for low-achieving youths, while DoD increased slightly for medium/high achieving and markedly for low-achieving youths, resulting in growing achievement-related disparities in both types of mortality. The trends were primarily driven by native-born youth and girls.

**Conclusions:**

The trends in Swedish youths resemble the trends in the American working-age population, but partly contrasts with corresponding trends in other European countries. Future research should investigate if the underlying causes that have been emphasized in the American context – socio-economic transformations and a greater supply of drugs – have also contributed to the Swedish trends.

## Highlights

• Paper studies trends in mortality and deaths of despair in Swedish youths 1990–2018.
• Finds rising rates of deaths of despair among low-achieving youths.
• Finds growing educational inequalities in mortality and deaths of despair.
• Trends driven by native-born youths and girls.
• Swedish trends resemble American trends but partly deviate from European ones.

## 1 Introduction

Rising rates of mortality due to suicide, drug overdose, and alcohol-related liver disease in the United States have received growing attention in both research and public debate (King et al., 2022). In a seminal paper, Case and Deaton (2017) used the umbrella term “deaths of despair” (DoD) to highlight the commonalities between these causes of death. In this context, “despair” is not used as a clinical or diagnostic term. Rather, deaths from suicide, drug- or alcohol-use are viewed as manifestations of an underlying sense of hopelessness, alienation, and lack of dignity, which, in turn, reflects a broader societal malaise characterized by diminishing employment prospects and a disintegration of community life (Case & Deaton, 2022; Copeland et al., 2020; Glei et al., 2024; Zheng & Choi, 2024).

At the core of the notion of DoD is that despair is socially stratified, with education being placed in the foreground from the onset (King et al., 2022). In the United States, the economic and social drivers of despair have primarily affected the less educated and resulted in a widening educational gradient in deaths from suicide and drug- or alcohol-use (Case & Deaton, 2022). In addition to education, race and gender has been highlighted as stratifying dimensions. The rise in DoD has particularly impacted white Americans, such that DoD-rates among whites now surpass those of any other racial group save for Native Americans (Tilstra et al., 2021; Woolf et al., 2018). Regarding gender, DoD-rates are higher in American men, but, contrary to widespread miconceptions (Case & Deaton, 2022), the increase over time in DoD-rates as well as in the educational gradient are similar for women (Case & Deaton, 2017; Tilstra et al., 2021).

The present study investigated trends in the association between academic achievement and all-cause mortality (ACM) and DoD in Swedish youths. It thereby makes three key contributions to the existing literature on DoD. First, subsequent research has moved beyond the American context and found increases in DoD in other national contexts, such as in the United Kingdom (Allik et al., 2020; Augarde et al., 2022) and in Canada (Dowd et al., 2023). Yet, research on temporal trends from countries outside of the Anglosphere are largely lacking, prompting calls for research from more diverse geographical contexts (King et al., 2022). Here, the Swedish case is particularly relevant given that mortality trends in Swedish young adults (aged 20–34) after the year 2000 diverged from Western Europe as a whole (Ågren & Bremberg, 2022). The divergence was partly driven by DoD, with a substantial increase in overdoses and a non-decline in suicides in Sweden, which contrasted with a reduction in both overdoses and suicides in Western Europe at large. Using more recent data, the Public Health Agency of Sweden (2023) has found rising suicide rates in Swedish youths aged 20–29 years, in contrast to generally declining rates in older age groups.

Second, there is a lack of studies on educational inequalities in trends in DoD outside of the United States, which is problematic given the centrality of education in the notion of DoD (Case & Deaton, 2022). For instance, Ågren and Bremberg (2022) proposed that the adverse mortality trends among Swedish young adults may be due to rising rates of school failure but their reliance on aggregate mortality data precluded an empirical analysis of such educational differences.

Third, youth has largely remained a blind spot in the literature on DoD (Benny et al., 2023), with no study to date investigating educational differences in DoD-trends among youths. However, DoD is of particular relevance in the context of youths. Although all-cause mortality increases strongly with age, youths are disproportionately likely to die from suicide and overdoses (Ward et al., 2021), meaning that changes in DoD may have a disproportionate impact on ACM-trends among youths. In addition, suicides and overdoses are directly linked to mood and substance use disorders (Edwards et al., 2020; Na et al., 2022; Volkow, 2004). Thus, a greater understanding of trends in DoD among youths may provide insights into related, and worrying, trends in mental ill-health among youths (Armitage et al., 2024).

### 1.1 Study setting

Compulsory school in Sweden spans from ages 6 to 16 (school year 9), with graduation from this stage corresponding to attaining an International Standard Classification of Education (ISCED) level 2. During compulsory school, students receive grades in 17 required subjects. Grades are assigned locally by schools but harmonized nationally through standardization (years 1990–1997) or national criteria and tests (1998–2010). At graduation, the subject grades are combined into an average grade (1990–1997) or a grade sum (1998–2010); henceforth referred to as a grade point average (GPA). After compulsory school, students can attend a three-year upper secondary school that provides a degree corresponding to ISCED level 3. Although optional, close to a 100% of students enrol at least the first year (Halapuu, 2021). Over the studied period, the stakes attached to students’ compulsory school grades became higher with schools increasingly selecting applicants solely based on compulsory school GPA (Swedish National Audit Office, 2018). Moreover, eligibility requirements were introduced in 1998, precluding students who did not achieve a minimum threshold of passing grades from attending.

### 1.2 Aim

The aim this study was to investigate changes in the association between academic achievement in compulsory school 1990–2010 and subsequent ACM and DoD between 1990 and 2018 in Swedish youths, as well as differences in these changes by sex and country of birth.

## 2 Methods

### 2.1 Data and participants

Individual-level data from Swedish administrative register were used in the analyses. Data on deaths were taken from the National Cause of Death Register, which includes data on more than 99% of all deaths in Sweden, including deaths of Swedish residents occurring abroad (National Board of Health and Welfare, 2022a). Data on compulsory school achievement and additional data on background variables were obtained from Statistics Sweden, 2024a, Statistics Sweden, 2024b.

All students that graduated from year 9 of compulsory school between 1990 and 2010 were included in the analysis. Regardless of graduation year, participants were followed for a maximum of eight years, starting from the year after graduation, or until emigration or death. Since 99.9% of the participants were between 15 and 17 years at the time of graduation, most were followed until age 23–25. An eight-year follow-up window was chosen since a majority of Swedish youth have entered the labor market by that age (Fondberg & Kreuger Wikmark, 2023). Thus, insofar as low compulsory school achievements constrain subsequent employment opportunities, most youth of that age will have faced these constraints. The sample size with complete data on academic achievement was 2 252 938. The data were made available for research by the Umeå SIMSAM Lab (Lindgren et al., 2016).

### 2.2 Outcomes: ACM and DoD

ACM was measured as death from any cause. We strove to follow established practice in the literature on DoD (Case & Deaton, 2017; Knapp et al., 2019) and used the following International Classification of Diseases (ICD) 10 or 9 codes to indicate DoD:

Suicide: Intentional self-harm and sequelae of intentional self-harm (ICD10: X60–X84, Y87.0; ICD9: E950–E959).

Drug overdose: Poisoning by and exposure to drugs and alcohol, whether accidental (ICD10: X40–X45; ICD9: E850–E855, E858, E860), with undetermined intent (ICD10: Y10–Y15; ICD9: E980.0–E980.5), or due to adverse effects in therapeutic use (ICD10: Y45, Y47, Y49; ICD9: E935, E937, E939).

Alcohol–related liver disease: Alcoholic liver disease, chronic hepatitis, and fibrosis and cirrhosis of liver (ICD10: K70, K73, K74; ICD9: 571).

Only the underlying cause of death was used.

### 2.3 Exposure: academic achievement

**Fig. 1:**
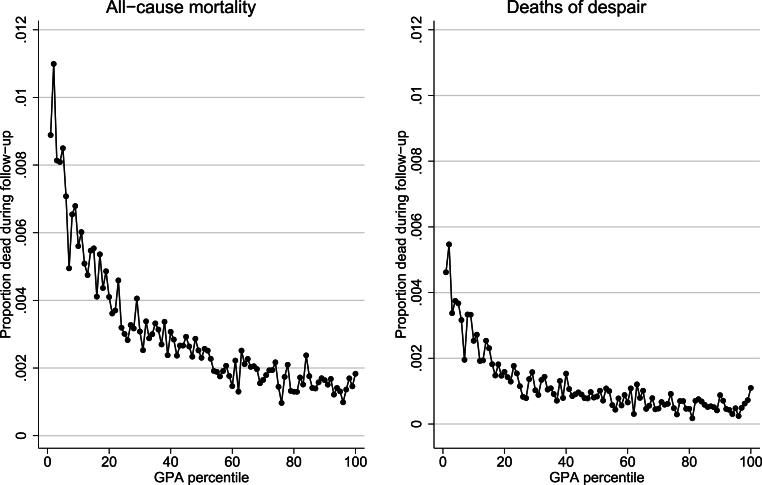
Average risk of all-cause mortality and deaths of despair by GPA percentile Dots indicate the predicted risk of all-cause mortality or deaths of despair in each GPA percentile. The predicted risks are derived from a discrete time proportional hazard models with all-cause mortality or deaths of despair as the outcome and GPA percentiles as the sole independent variable. GPA percentiles were entered as categorical variables with one category for each percentile, and with the first percentile as reference category. The predicted risks are calculated for all graduation cohorts combined.Abbreviations: GPA = Grade point average.

Academic achievement was measured by students’ final year GPA, with GPA transformed into percentile ranks within each graduation year to account for grade inflation and changes in the grading system during the studied period. The percentiles were then categorized into low (0^th^–20th percentiles) and medium/high GPA (21st–100th percentiles). The categorization was due to there being a clear non-linear association between achievement and both ACM and DoD, with youth in the approximately bottom 20% of the GPA distribution having markedly higher risks than their higher achieving peers (see Fig. 1 below). Thus, the categorization aimed to direct the analysis towards trends in the most vulnerable segment of the youth population.

### 2.4 Moderator: graduation year

Graduation year was defined as the first year that a youth received a final end-of-the year grade upon completing compulsory school. To enable more precise estimates within subgroups, graduation years were in some analyses grouped into three broader categories: 1990–1997, 1998–2004 and 2005–2010. The cut-off after 1997 was motivated by the fact that 1998 was the first year with formal eligibility requirements in upper secondary school. The cut-off after 2004 was motivated by the aim to use approximately equally sized categories, thereby having comparable statistical power to detect significant effects.

### 2.5 Subgroups: sex, country of birth

Analyses were stratified by sex and country of birth. Sex was measured as legal sex (0 = boy; 1 = girl). Country of birth was categorized into native-born (=0) and foreign-born (=1).

### 2.6 Statistical analysis

In the first stage of the analysis, the proportion that died during follow-up from ACM or DoD was calculated in each single graduation year and GPA-group. ACM and DoD are rare events, especially in foreign-born students and girls. For instance, the number of foreign-born youths graduating in a given year that died from DoD ranged from 1 to 11 (medium/high GPA) or 2 to 10 (low GPA; see Supplementary file A, Tables A2 & A3). Three-year moving averages were therefore used to smooth out the resulting short-term fluctuations over time and make it easier to identify the underlying trends.

In the second stage, proportional hazard models were used to formally address the aim of the study. These models were chosen as they are specifically designed to analyze time-to-event data (i.e., time from graduation to death) while accounting for right-censoring due to e.g. emigration (Allison, 1982). Since only data on the year, not the exact date, of graduation or death were available, discrete time proportional hazard models, fitted using complementary log-log regression models, were used. The complementary log-log specification provides a discrete time representation of a continuous time proportional hazards model (Allison, 1982). The following complementary log-log model was estimated:

$$\ln\left(-\ln\left(1-P_{t}\right)\right)=\alpha_{0}+\beta_{1}GPA+\beta_{2}GradYear+\beta_{3}GPA*GradYear$$

where ln(−ln) is the logarithm of the negative log, $P_{t}$ is the probability of ACM, DoD, or non-despair related causes of death at the time interval t, $\alpha_{0}$ is the intercept, $\beta_{1}$ is the main effect of GPA, $\beta_{2}$ is the main effect of graduation year (the categorical indicator), and $\beta_{3}$ is the coefficient for the interaction between GPA and graduation year. $\beta_{3}$ formally tests the aim of the study: if it is significantly (p<0.05) different from 1, it is concluded that the association between academic achievement (GPA) and subsequent ACM and DoD changed across graduation years.

Separate models were fitted for ACM and DoD. In analyses of DoD, all other, non-despair related, causes of death constitute competing risks that prevent he focal event (DoD) from occurring. Cause-specific (i.e., competing risk) proportional hazard models were therefore fitted for DoD and non-DoD mortality, with units that experienced the competing event treated as censored in analyses of the focal event and vice versa (Lau et al., 2009). The models were first fitted on the full sample and then stratified by, respectively, sex and country of birth. A key assumption underlying discrete time proportional hazard models is the proportional hazard assumption, stating that the hazard ratios for the exposure and control groups are constant over follow-up time. Supplementary file B shows that this assumption was satisfied in the models.

A range of supplementary and sensitivity analyses were also conducted. Additional descriptive statistics on the sample is included in Supplementary file A. The sensitivity of the results to the operationalization of DoD was investigated by using a broader indicator of DoD, comprising deaths due to mental, behavioral and neurodevelopmental disorders (ICD10: F00–F99; ICD9: 290–319) as well as deaths from injuries of undetermined intent (ICD10: Y16–Y34; ICD9: E980–989). The latter set was used to account for possible misclassification of suicides (Gunnell et al., 2013). Moreover, due to criticism that the components of DoD are too diverse to be grouped into one category (Tilstra, 2023), the separate components were analysed separately.

The sensitivity of the results to the choice of operationalization of GPA was investigated by (i) using a continuous measure of GPA in place of the categorical one used in the main analyses; (ii) calculating GPA percentiles separately for Swedish- and foreign-born youths to account for possible demographic shifts in the composition of the low achieving group; and (iii) using stricter (1st–10th percentiles) and broader (1st–30th percentiles) categories to indicate low GPA.

The sensitivity of the results to the choice of operationalization of graduation years was investigated by replacing the categorical indicator with a continuous measure, and by presenting the proportion who died during follow-up per graduation year without using three-year moving averages. The sensitivity of the results to the operationalization of country of birth was investigated by using a more fine-grained measure, taking the country of birth of the participants as well as of their parents into account.

A key assumption underlying cause-specific competing risks models is that the competing events are independent of each other (Lau et al., 2009). We therefore also estimated subdistribution hazard models (Fine & Gray, 1999), which relax this assumption by keeping units experiencing the competing event in the risk set. Subdistribution hazard models require continuous time-to-event data but methods for continuous data can be used as an approximation for discrete data (Schmid & Berger, 2021).

The main manuscript investigated multiplicative interaction between GPA and graduation years, which is a relative measure of change. Two measures of additive interaction, which are absolute measures of change, were investigated in supplementary analyses: the relative excess risk due to interaction and the attributable proportion.

## 3 Results

Table 1 presents summary statistics on the full sample. There were 7007 deaths in total, and 2747 DoD, during follow-up. Close to three times more low-achieving than medium/high-achieving youths died from any cause, and more than three times more died from DoD. Note that the bottom quintile of the GPA distribution does not exactly equal 20% due to clustering of grades at certain cutoff values. ACM declined somewhat, while DoD increased somewhat, in later graduation years, meaning that a larger share of all deaths in later graduation years were due to DoD. Around 2.5 times more boys than girls died from ACM or DoD, while mortality differences depending on country of birth were small.

**Table 1:** Summary statistics.

	Number of individuals	% of sample	Number of deaths: ACM	% dead: ACM	Number of deaths: DoD	% dead: DoD
*GPA*						
Medium/high GPA	1 764 070	78.30 %	4005	0.23 %	1423	0.08 %
Low GPA	488 868	21.70 %	3002	0.61 %	1324	0.27 %
*Graduation year*						
1990–1997	798 545	35.44 %	2607	0.33 %	815	0.10 %
1998–2004	728 655	32.34 %	2330	0.32 %	915	0.13 %
2005–2010	725 738	32.21 %	2070	0.29 %	1017	0.14 %
*Sex*						
Boy	1 152 986	51.18 %	5102	0.44 %	2023	0.18 %
Girl	1 099 918	48.82 %	1905	0.17 %	724	0.07 %
*Country of birth*						
Sweden	2 077 701	92.22 %	6414	0.31 %	2530	0.12 %
Foreign-born	175 181	7.78 %	593	0.34 %	217	0.12 %
*Total*	2 252 938	100 %	7007	0.31 %	2747	0.12 %

Abbreviations: ACM = all-cause mortality; DoD = deaths of despair. % dead = percent dead during follow up.

Fig. 1 shows the average risks of ACM or DoD during follow-up per GPA percentile in all graduation years combined. As mentioned in section 2.3., there was a non-linear relationship between GPA and ACM or DoD, with high risks in the very lowest percentiles that declined sharply until approximately the 20th percentile, after which the decline leveled off. Thus, the lowest approximately 20th percentiles had disproportionately high risks, while the differences in the middle and higher end of the distribution were smaller.

**Fig. 2:**
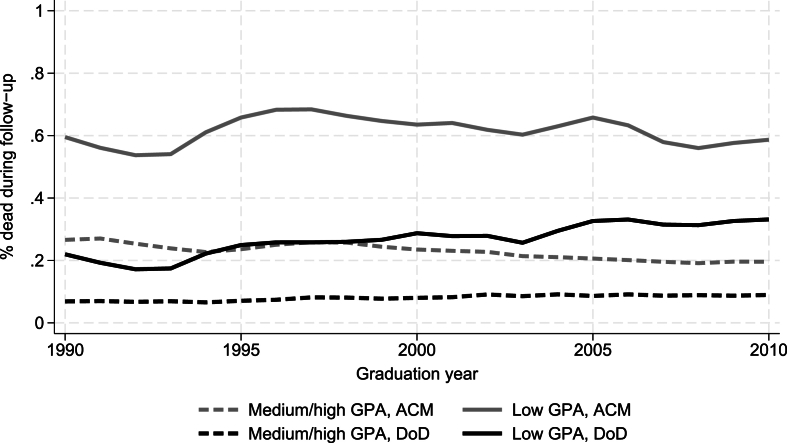
Proportion in % of youths that died during follow-up from all causes or from despair by GPA. Three-year moving averages. Abbreviations: ACM = all-cause mortality; DoD = Deaths of despair; GPA = Grade point average.

**Fig. 3:**
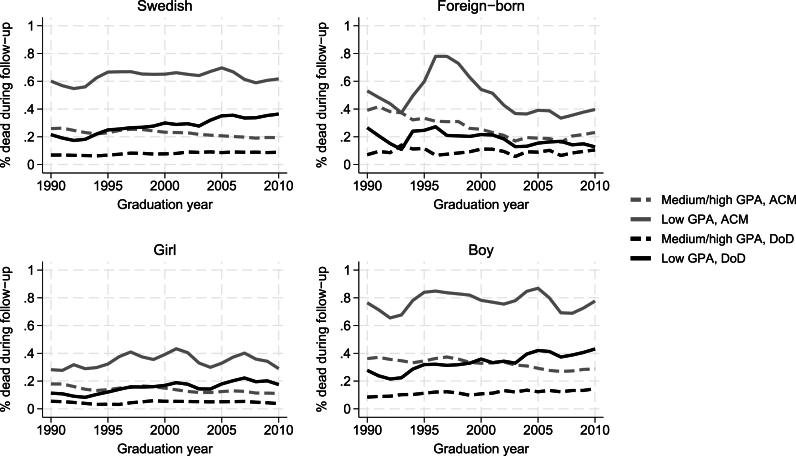
Proportion in % of youths that died during follow-up from all causes or from despair by GPA, country of birth and sex. Three-year moving averages. Abbreviations: ACM = all-cause mortality; DoD = Deaths of despair; GPA = Grade point average.

Fig. 2, Fig. 3 show the percent (y-axis) in each graduation year (x-axis) that died during follow-up from ACM (grey lines) or from DoD (black lines). In the full sample (Fig. 2), ACM-rates declined in medium/high-achieving youths (21st-100th GPA percentile) but remained largely stable in low-achieving youths (1st – 20th GPA percentiles). DoD-rates increased slightly in medium/high-achieving youths and more markedly in low-achieving youths. Fig. 3 shows that the increase in DoD was even more marked for low-achieving Swedish youths, while the trend for low-achieving foreign-born youth was less consistent but if anything points toward a downward trend in DoD-rates. The “bumpiness” of the lines for foreign-born youths reflects the smaller samples in this group (see Supplementary file A, Table A2). DoD-rates increased in both low-achieving girls and boys, but from a lower baseline level in girls.

Table 2, Table 3 formally address the study's aim, with Table 2 presenting results with ACM as the outcome. The first column shows that there was a positive association between low achievement and ACM already in 1990–1997 (the reference category), and that there was a negative association between graduation year and ACM in medium/high achieving youths (the reference category). In other words, ACM declined for medium/high achieving youths who graduated after 1990–1997. The interaction terms show that the positive association between low achievement and ACM became stronger for those graduating after 1990–1997. Since hazard ratios are multiplicative, the interaction terms imply that ACM remained stable in low-achieving youths (1.127 X 0.919 = 1.036; 0.795 X 1.253 = 0.996). The stratified models show that the association between low achievement and ACM became stronger over time in native-born but not foreign-born youths, and in both girls and boys (only boys graduating in 2005–2010).

**Table 2:** Discrete time proportional hazard models with all-cause mortality as the outcome.

	All	Native-born	Foreign-born	Girls	Boys
*GPA (ref: medium/high GPA)*					
Low GPA	2.434∗∗∗	2.502∗∗∗	1.686∗∗∗	2.075∗∗∗	2.149∗∗∗
	[2.250,2.632]	[2.304,2.718]	[1.307,2.176]	[1.757,2.450]	[1.963,2.352]
*Graduation year (ref: 1990–1997)*					
1998–2004	0.919∗	0.945	0.636∗∗∗	0.892	0.924
	[0.854,0.989]	[0.876,1.021]	[0.490,0.826]	[0.783,1.017]	[0.845,1.010]
2005–2010	0.795∗∗∗	0.816∗∗∗	0.569∗∗∗	0.788∗∗∗	0.788∗∗∗
	[0.737,0.858]	[0.754,0.883]	[0.423,0.766]	[0.688,0.902]	[0.718,0.864]
*Graduation year X GPA*					
1998-2004 X Low GPA	1.127∗	1.131∗	1.307	1.273∗	1.126
	[1.006,1.262]	[1.004,1.274]	[0.902,1.893]	[1.009,1.606]	[0.988,1.285]
2005-2010 X Low GPA	1.253∗∗∗	1.282∗∗∗	1.141	1.420∗∗	1.271∗∗∗
	[1.115,1.408]	[1.135,1.448]	[0.752,1.734]	[1.121,1.800]	[1.109,1.456]
N (individuals)	2 252 938	2 077 701	175 181	1 099 918	1 152 986
N (observations)	17 791 418	16 437 773	1 353 255	8 666 587	9 124 584

Table reports hazard ratios, with 95% confidence intervals in brackets. ∗p<0.05, ∗∗p<0.01, ∗∗∗p<0.001. Abbreviations: ref = reference category; GPA = Grade point average.

**Table 3:** Cause-specific discrete time proportional hazard models with deaths of despair and all other causes of death as outcomes.

	All	Native-born	Foreign-born	Girls	Boys
**Outcome: Deaths of despair**					
*GPA (ref: medium/high GPA)*					
Low GPA	3.073∗∗∗	3.134∗∗∗	2.319∗∗∗	2.770∗∗∗	2.635∗∗∗
	[2.677,3.527]	[2.711,3.622]	[1.469,3.660]	[2.066,3.714]	[2.249,3.086]
*Graduation year (ref: 1990–1997)*					
1998–2004	1.174∗	1.196∗∗	0.906	1.332∗	1.097
	[1.031,1.336]	[1.044,1.369]	[0.570,1.438]	[1.058,1.676]	[0.937,1.283]
2005–2010	1.260∗∗∗	1.278∗∗∗	1.057	1.185	1.273∗∗
	[1.110,1.431]	[1.120,1.458]	[0.655,1.707]	[0.935,1.501]	[1.095,1.481]
*Graduation year X GPA*					
1998-2004 X Low GPA	1.067	1.101	0.941	1.088	1.131
	[0.883,1.289]	[0.903,1.343]	[0.500,1.774]	[0.740,1.598]	[0.908,1.410]
2005-2010 X Low GPA	1.202	1.274∗	0.688	1.521∗	1.176
	[0.999,1.446]	[1.051,1.545]	[0.351,1.348]	[1.040,2.225]	[0.951,1.456]
**Outcome: Other causes of death**					
*GPA (ref: medium/high GPA)*					
Low GPA	2.181∗∗∗	2.251∗∗∗	1.458∗	1.827∗∗∗	1.947∗∗∗
	[1.981,2.400]	[2.035,2.490]	[1.069,1.988]	[1.491,2.238]	[1.743,2.175]
*Graduation year (ref: 1990–1997)*					
1998–2004	0.819∗∗∗	0.846∗∗∗	0.539∗∗∗	0.736∗∗∗	0.852∗∗
	[0.749,0.896]	[0.770,0.929]	[0.391,0.742]	[0.626,0.864]	[0.765,0.950]
2005–2010	0.612∗∗∗	0.632∗∗∗	0.393∗∗∗	0.647∗∗∗	0.587∗∗∗
	[0.555,0.674]	[0.571,0.699]	[0.265,0.584]	[0.547,0.764]	[0.520,0.662]
*Graduation year X GPA*					
1998-2004 X Low GPA	1.118	1.101	1.520	1.314	1.090
	[0.969,1.289]	[0.947,1.280]	[0.959,2.410]	[0.978,1.766]	[0.924,1.285]
2005-2010 X Low GPA	1.158	1.144	1.539	1.160	1.232∗
	[0.991,1.353]	[0.971,1.347]	[0.892,2.657]	[0.846,1.592]	[1.027,1.478]
N (individuals)	2 252 938	2 077 701	175 181	1 099 918	1 152 986
N (observations)	17 791 418	16 437 773	1 353 255	8 666 587	9 124 584

Table reports hazard ratios, with 95% confidence intervals in brackets. ∗p<0.05, ∗∗p<0.01, ∗∗∗p<0.001. Abbreviations: ref = reference category; GPA = Grade point average.

Table 3 presents results with DoD (top rows) as well non-DoD related mortality (bottoms rows) as the outcomes. There was a positive association between graduation year and DoD in medium/high achieving youths in the full sample, in native-born youths and in girls (graduating 1998–2004) and boys (graduating 2005–2010). The interaction terms show that the positive association between low achievement and DoD did not change across graduation years in the full sample, while the stratified analysis show that it became stronger for native-born youths and for girls graduating in 2005–2010. Thus, the increase in DoD observed for medium/high achieving youths in these groups was even larger for low-achieving youths. Since hazard ratios are multiplicative, the risks for low-achieving native-born youths and girls graduating in 2005–2010 were, respectively, 1.628 (1.278 X 1.274) and 1.802 (1.185 X 1.521) times higher than for those graduating in 1990–1997. The association between low achievement and other causes of death (bottoms rows) did not change across graduation years for the full sample, and only for boys in the stratified analyses.

### 3.1 Supplementary analyses

The results were qualitatively similar when using a broader indicator of DoD (Supplementary file C), alternative operationalizations of GPA (Supplementary file D) and graduation years (Supplementary file E), and subdistribution hazard models (Supplementary file F). In addition, Supplementary file C shows that the interaction between GPA and graduation year was not significant with the respective components of DoD (suicide and drug overdoses) as outcomes, probably due to smaller samples. Supplementary file G shows that measures of additive interaction were greater for DoD than for ACM, and especially pronounced for native-born youth and girls. For instance, 41% of DoD in low-achieving girls, and 33% of DoD in low-achieving native-born youths, who graduated in 2005–2010 could be attributed to the interaction between GPA and graduation year. Supplementary file H shows that the increase in the achievement-related disparities were primarily driven by native-born youths with native-born parents.

## 4 Discussion

The aim of this study was to investigate changes in the association between academic achievement in compulsory school 1990–2010 and subsequent ACM and DoD between 1990 and 2018 in Swedish youths, as well as differences in these changes by sex and country of birth. We highlight three key findings. First, ACM declined in medium/high-achieving but not in low-achieving youths, resulting in growing educational inequalities in ACM over time. Second, stratified analyses showed that these growing inequalities were primarily driven by native-born youths and girls, with a less consistent trend in boys and no clear trend in foreign-born students. Third, while DoD increased in both medium/high- and low-achieving youths in the full sample, stratified analyses showed that the increase was larger in low-achieving native-born youths and girls, resulting in growing educational inequalities in DoD in these groups.

There are some striking similarities between these findings and corresponding temporal trends in the American working-age population that has been the focus in the existing literature on DoD. First, ACM in working-age Americans has been largely stable or only increased slightly on average (Case & Deaton, 2017; Woolf et al., 2018), but this stability masks substantial educational disparities, with declining mortality in the higher and growing mortality in the lower educated (Case & Deaton, 2017). Our findings partly align with this trend. While ACM *declined* in medium/high-achieving youths, it was *stable* among low-achieving youths, resulting in growing educational inequalities. Second, while DoD has increased on average in working-age Americans (Dowd et al., 2023; Tilstra et al., 2021), the increase has been largest among the lower educated (Case & Deaton, 2017, 2022; Ho, 2017). This is in line with our findings, at least for native-born youths and girls, and in both cases the result has been in growing educational inequalities in DoD. Third, the increase in ACM among the lower educated in the US has been concentrated to non-Hispanic whites (Case & Deaton, 2017). Albeit race or ethnicity is not recorded in Swedish registers, it is notable that DoD increased in low-achieving native-born but not foreign-born youths.

Our results are only partly consistent with mortality trends in the European working-age population. While ACM has generally declined among both the lower and higher educated, relative inequalities have increased but absolute inequalities have been reduced (Mackenbach et al., 2018). In contrast, we found that ACM declined in medium/high-achieving but not low-achieving youths, resulting in larger relative *and* absolute inequalities. We are not aware of any research on temporal trends or educational inequalities in DoD *per se* among youths outside of the USA. However, research on the specific components shows that youth suicide has declined in Europe but been stable or increased slightly in Sweden (Bertuccio et al., 2024; Public Health Agency of Sweden, 2023), while drug overdoses in young adults has declined in Western Europe but increased in Sweden (Ågren & Bremberg, 2022). Findings on broader socio-economic inequalities among adult Europeans are mixed, with some studies showing increasing inequalities in drug-related but not alcohol-related mortality or suicides (Allik et al., 2020), and others, including Swedish ones, showing increasing inequalities in suicide and alcohol-related mortality in women but no or smaller increases in men (Budhiraja & Landberg, 2015; Hiyoshi et al., 2018; Lorant et al., 2018). The latter is partly in line with our finding that the greatest relative increase in DoD was observed in low-achieving girls. Going beyond mortality, our findings are also consistent with research showing growing achievement-related inequalities in attempted suicide and psychiatric conditions in Sweden (Högberg et al., 2024; Sörberg Wallin et al., 2020).

The present study was descriptive, and we did not attempt to investigate the causes of the observed trends. The primarily American literature on trends in DoD has been centred around demand- or supply-side factors (Bjorklund, 2023; King et al., 2022). Demand-side explanations focus on socio-economic transformations – such as deindustrialization, deteriorating employment prospects, and economic inequality – that, in turn, have contributed to social disintegration and growing sense of hopelessness, primarily among the lower educated (Autor et al., 2019; Bjorklund, 2023; Case & Deaton, 2022; Dow et al., 2020; Fishman & Gutin, 2021; Knapp et al., 2019; Kuo & Kawachi, 2023; Pierce & Schott, 2020; Rambotti, 2020; Wu & Evangelist, 2022). Supply-side explanations focus on the fact that the increase in DoD in Anglo-Saxon countries has primarily been driven by drugs rather than suicides or alcohol (Allik et al., 2020; Dowd et al., 2023; Tilstra et al., 2021), and that the main driver of these drug deaths is a greater supply of prescribed or illicit drugs such as synthetic opioids (Ruhm, 2022; Thombs et al., 2020).

Both explanations may have bearing on the Swedish context. As for demand-side explanations, the Swedish occupational structure has been transformed, with a decline in routine and industrial jobs (Oesch & Piccitto, 2019). Meanwhile, the average level of education in the labor force has increased faster than the skill-requirements of available jobs, leading to overeducation (Korpi & Tåhlin, 2009). Taken together, these processes imply that lower educated workers increasingly compete with higher educated workers for a diminishing number of lower-skilled jobs; a competition in which the lower educated workers, and *á fortiori*, lower educated young workers, are bound to come up short (Tåhlin & Westerman, 2020). However, the follow-up period covered in our study includes ages when most Swedish youth have not yet entered the labor market. A potential demand-side explanation that has bearing on the school-age years, and that is partly unique to the Swedish education system, is the strict eligibility requirements that were introduced in 1998 and that since then have excluded 10–15% of graduates each year from the regular upper secondary school. Evidence shows that the eligibility requirements have resulted in lower educational attainment, employment and incomes, and a greater uptake of disability and sickness benefits, among excluded students (Diamond & Persson, 2016; Halapuu, 2021).

As for supply-side explanations, the street price of illicit drugs in Sweden has decreased since the early 1990s and the consumption of such drugs among Swedish youths has increased (The Swedish Council for Information on Alcohol and Other Drugs, 2017). Moreover, the “epidemic” of synthetic opioids such as fentanyl, notorious in the USA, has left a mark on Swedish mortality trends as well (National Board of Health and Welfare, 2022b).

A third potential explanation, beyond demand- and supply-side factors, is that the composition of the low-achieving group has changed over time. It is well-established that substance abuse and other health conditions can undermine academic achievement (Esch et al., 2014). If youths with such problems, for whatever reason, become increasingly concentrated in the lower end of the achievement distribution, we would expect a stronger association between achievement and ACM or DoD over time. This explanation – stronger health selection over time – has seldom been considered in the literature on DoD but is prominent in the broader literature on health inequalities.

### 4.1 Limitations

The notion of DoD has been criticized for grouping together disparate causes of deaths that have little in common in terms of aetiology (Dowd et al., 2023; Ruhm, 2022; Shanahan & Copeland, 2021; Shanahan et al., 2019; Tilstra, 2023; Tilstra et al., 2021). When analyzing suicides and drug overdoses separately, there was no significant change in the association between achievement and mortality, probably because of the fewer number of deaths in each separate category. Thus, the veracity of our findings partly hinges upon whether or not the broader DoD-category itself is deemed meaningful. Related to this, we did not have access to some possibly relevant causes of death – for example, ICD 10-codes K85.2 and K86.0 (alcohol-related pancreatitis) – that have been used in previous research on DoD (Augarde et al., 2022; Lübker & Murtin, 2024). Another limitation concerns the operationalization of achievement. We relied on teacher-assigned grades as a measure of achievement, which, since grading practices vary across Swedish schools (Diamond & Persson, 2016), may intrododuce measurement error. Lastly, much of the American literature on DoD has focused on racial disparities (Case & Deaton, 2022; King et al., 2022; Tilstra et al., 2021). Race is not recorded in Swedish registers, and our results concerning country of birth cannot be directly compared to American findings on racial differences. Importantly, the composition of the foreign-born group changed over the studied period, with foreign-born students graduating after the 1990s arriving at a higher age on average and coming from origin countries with a lower level of social and economic development (Swedish National Agency for Education, 2016). Changes in the composition of the low-achieving foreign-born group complicate interpretations of the trends in this group.

## 5 Conclusions

This study found stagnating trends in ACM and a rise in DoD among low-achieving youths in Sweden, resulting in growing educational inequalities in mortality, especially in girls and native-born youths. These worrying trends suggests that mortality, and not least DoD, in low-achieving students and lower educated youths should be a public health priority. The trends in Swedish youths resemble the trends in the American working-age population, but partly contrasts with European trends toward declining ACM in both lower and higher educated adults as well as declining levels of drug overdoses suicides in youths. It is not clear why the situation for Swedish youths resembles the American situation more than that in other European countries. Future research should investigate if causes that have been highlighted in the American context – socio-economic transformations and a greater supply of drugs, but also stronger health selection over time – have also contributed to the Swedish trends. Future research should also investigate other causes of death besides DoD that may have contributed to the widening educational inequalities in youth mortality.

## CRediT authorship contribution statement

**Björn Högberg:** Writing – review & editing, Writing – original draft, Project administration, Methodology, Investigation, Funding acquisition, Formal analysis, Data curation, Conceptualization. **Simone Scarpa:** Writing – review & editing, Validation. **Solveig Petersen:** Writing – review & editing, Validation.

## Ethical statement

The use of data for this study was approved by the Swedish Ethical Review Authority (Dnr 2023-03999-01). The study was based on register data. Informed consent of participation is therefore not applicable.

## Declaration of competing interest

The authors declare the following financial interests/personal relationships which may be considered as potential competing interests: Bjorn Hogberg reports financial support was provided by 10.13039/501100006636Swedish Research Council for Health, Working Life and Welfare. If there are other authors, they declare that they have no known competing financial interests or personal relationships that could have appeared to influence the work reported in this paper.

## Data Availability

The authors do not have permission to share data.
