# Supplementary material for: Trends in educational inequalities in all-course mortality and deaths of despair in Swedish youths 1990–2018
Source: SSM Popul Health. 2025 Jan 4;29:101748. doi: 10.1016/j.ssmph.2025.101748 (PMC11780141; doi:10.1016/j.ssmph.2025.101748)
Supplement: Multimedia component 1 [file mmc1.docx]

**Supplementary materials for *Growing educational inequalities in all-course mortality and deaths of despair in Swedish youths 1990-2018***

**Supplementary file A. Additional descriptive statistics**

**Supplementary file B. Log-log plots**

**Supplementary file C. Alternative operationalizations of deaths of despair**

**Supplementary file D. Alternative operationalizations of achievement**

**Supplementary file E. Alternative operationalizations of graduation year**

**Supplementary file F. Subdistribution hazard models**

**Supplementary file G. Additive interactions**

**Supplementary file H. Alternative measures of country of birth**

Note that results for non-despair-related causes of death are not shown in the supplementary analyses for brevity and since they are not in focus of the study.

**Supplementary file A. Additional descriptive statistics**

**Table A1. Sample size and number of deaths by GPA and graduation year**

|  | *Number of individuals* | | *All-cause mortality* | | *Deaths of despair* | |
| --- | --- | --- | --- | --- | --- | --- |
| *Graduation year* | *Medium/*  *high GPA* | *Low GPA* | *Medium/*  *high GPA* | *Low GPA* | *Medium/*  *high GPA* | *Low GPA* |
| 1990 | 85597 | 23864 | 228 | 146 | 56 | 60 |
| 1991 | 81743 | 21664 | 217 | 125 | 59 | 40 |
| 1992 | 77969 | 20448 | 218 | 99 | 56 | 27 |
| 1993 | 76425 | 20819 | 164 | 114 | 44 | 41 |
| 1994 | 73912 | 20709 | 163 | 122 | 58 | 40 |
| 1995 | 76681 | 21513 | 188 | 149 | 47 | 59 |
| 1996 | 78707 | 21156 | 190 | 146 | 57 | 59 |
| 1997 | 77182 | 20156 | 204 | 134 | 68 | 44 |
| 1998 | 74809 | 22278 | 202 | 155 | 63 | 61 |
| 1999 | 74307 | 22246 | 176 | 140 | 52 | 63 |
| 2000 | 76840 | 22729 | 173 | 140 | 60 | 55 |
| 2001 | 79978 | 24298 | 194 | 160 | 73 | 81 |
| 2002 | 82789 | 23863 | 186 | 154 | 64 | 61 |
| 2003 | 84841 | 23953 | 183 | 132 | 88 | 59 |
| 2004 | 91063 | 24661 | 184 | 151 | 69 | 66 |
| 2005 | 93579 | 25688 | 200 | 185 | 89 | 94 |
| 2006 | 99403 | 26562 | 201 | 170 | 87 | 91 |
| 2007 | 98846 | 26270 | 186 | 142 | 90 | 75 |
| 2008 | 98160 | 24686 | 193 | 137 | 81 | 78 |
| 2009 | 90531 | 27973 | 171 | 163 | 84 | 94 |
| 2010 | 90708 | 23332 | 184 | 138 | 78 | 76 |

**Table A2. Sample size and number of deaths by GPA, graduation year and country of birth**

|  | *Number of individuals* | | *All-cause mortality* | | *Deaths of despair* | |
| --- | --- | --- | --- | --- | --- | --- |
| *Graduation year* | *Medium/*  *high GPA* | *Low GPA* | *Medium/*  *high GPA* | *Low GPA* | *Medium/*  *high GPA* | *Low GPA* |
| *Native-born* |  |  |  |  |  |  |
| 1990 | 81454 | 21922 | 216 | 136 | 54 | 54 |
| 1991 | 77450 | 19836 | 196 | 115 | 55 | 36 |
| 1992 | 73687 | 18427 | 198 | 91 | 50 | 25 |
| 1993 | 72149 | 18742 | 156 | 106 | 43 | 38 |
| 1994 | 69598 | 18378 | 143 | 114 | 47 | 38 |
| 1995 | 71923 | 18849 | 173 | 130 | 44 | 47 |
| 1996 | 73080 | 18449 | 176 | 127 | 54 | 54 |
| 1997 | 71218 | 17441 | 182 | 109 | 63 | 39 |
| 1998 | 68932 | 19100 | 184 | 132 | 58 | 53 |
| 1999 | 68093 | 18945 | 160 | 121 | 47 | 57 |
| 2000 | 70508 | 19333 | 159 | 120 | 53 | 49 |
| 2001 | 73655 | 20824 | 176 | 144 | 64 | 71 |
| 2002 | 76400 | 20423 | 174 | 137 | 59 | 55 |
| 2003 | 78502 | 20576 | 173 | 121 | 84 | 56 |
| 2004 | 84934 | 21405 | 174 | 142 | 67 | 62 |
| 2005 | 87559 | 22441 | 184 | 169 | 78 | 88 |
| 2006 | 93634 | 23340 | 193 | 157 | 84 | 86 |
| 2007 | 93036 | 22922 | 177 | 133 | 86 | 70 |
| 2008 | 92892 | 21692 | 183 | 127 | 77 | 72 |
| 2009 | 86039 | 24467 | 158 | 147 | 79 | 91 |
| 2010 | 85677 | 19769 | 175 | 126 | 73 | 70 |
| *Foreign-born* |  |  |  |  |  |  |
| 1990 | 4142 | 1942 | 12 | 10 | 2 | 6 |
| 1991 | 4291 | 1827 | 21 | 10 | 4 | 4 |
| 1992 | 4280 | 2020 | 20 | 8 | 6 | 2 |
| 1993 | 4276 | 2076 | 8 | 8 | 1 | 3 |
| 1994 | 4311 | 2331 | 20 | 8 | 11 | 2 |
| 1995 | 4758 | 2664 | 15 | 19 | 3 | 12 |
| 1996 | 5626 | 2706 | 14 | 19 | 3 | 5 |
| 1997 | 5963 | 2713 | 22 | 25 | 5 | 5 |
| 1998 | 5877 | 3176 | 18 | 23 | 5 | 8 |
| 1999 | 6212 | 3299 | 16 | 19 | 5 | 6 |
| 2000 | 6332 | 3396 | 14 | 20 | 7 | 6 |
| 2001 | 6323 | 3474 | 18 | 16 | 9 | 10 |
| 2002 | 6389 | 3440 | 12 | 17 | 5 | 6 |
| 2003 | 6337 | 3375 | 10 | 11 | 4 | 3 |
| 2004 | 6127 | 3254 | 10 | 9 | 2 | 4 |
| 2005 | 6018 | 3246 | 16 | 16 | 11 | 6 |
| 2006 | 5768 | 3220 | 8 | 13 | 3 | 5 |
| 2007 | 5807 | 3347 | 9 | 9 | 4 | 5 |
| 2008 | 5264 | 2994 | 10 | 10 | 4 | 6 |
| 2009 | 4490 | 3501 | 13 | 16 | 5 | 3 |
| 2010 | 5029 | 3560 | 9 | 12 | 5 | 6 |

**Table A3. Sample size and number of deaths by GPA, graduation year and sex**

|  | *Number of individuals* | | *All-cause mortality* | | *Deaths of despair* | |
| --- | --- | --- | --- | --- | --- | --- |
| *Graduation year* | *Medium/*  *high GPA* | *Low GPA* | *Medium/*  *high GPA* | *Low GPA* | *Medium/*  *high GPA* | *Low GPA* |
| *Girls* |  |  |  |  |  |  |
| 1990 | 45023 | 8422 | 80 | 21 | 23 | 12 |
| 1991 | 43044 | 7527 | 78 | 24 | 25 | 6 |
| 1992 | 40923 | 7159 | 73 | 19 | 19 | 7 |
| 1993 | 39895 | 7336 | 48 | 27 | 13 | 7 |
| 1994 | 39006 | 7278 | 48 | 17 | 15 | 4 |
| 1995 | 40470 | 7592 | 62 | 22 | 10 | 12 |
| 1996 | 41509 | 7433 | 57 | 33 | 15 | 11 |
| 1997 | 40619 | 7024 | 64 | 28 | 14 | 8 |
| 1998 | 39033 | 8245 | 66 | 32 | 24 | 17 |
| 1999 | 38638 | 8252 | 65 | 28 | 22 | 12 |
| 2000 | 40043 | 8520 | 57 | 29 | 23 | 11 |
| 2001 | 41529 | 9274 | 57 | 45 | 20 | 21 |
| 2002 | 42865 | 9244 | 57 | 43 | 24 | 19 |
| 2003 | 43945 | 9611 | 47 | 26 | 24 | 10 |
| 2004 | 46735 | 9616 | 54 | 25 | 21 | 12 |
| 2005 | 48063 | 10198 | 62 | 37 | 25 | 20 |
| 2006 | 51339 | 10251 | 66 | 37 | 29 | 22 |
| 2007 | 50471 | 10139 | 66 | 40 | 24 | 20 |
| 2008 | 50159 | 9740 | 59 | 44 | 28 | 25 |
| 2009 | 47080 | 10835 | 43 | 26 | 18 | 15 |
| 2010 | 46658 | 9175 | 61 | 32 | 17 | 20 |
| *Boys* |  |  |  |  |  |  |
| 1990 | 40574 | 15442 | 148 | 125 | 33 | 48 |
| 1991 | 38698 | 14136 | 139 | 101 | 34 | 34 |
| 1992 | 37045 | 13289 | 145 | 80 | 37 | 20 |
| 1993 | 36530 | 13483 | 116 | 87 | 31 | 34 |
| 1994 | 34904 | 13431 | 115 | 105 | 43 | 36 |
| 1995 | 36211 | 13921 | 126 | 127 | 37 | 47 |
| 1996 | 37197 | 13722 | 133 | 113 | 42 | 48 |
| 1997 | 36563 | 13131 | 140 | 106 | 54 | 36 |
| 1998 | 35776 | 14031 | 136 | 123 | 39 | 44 |
| 1999 | 35667 | 13992 | 111 | 112 | 30 | 51 |
| 2000 | 36797 | 14209 | 116 | 111 | 37 | 44 |
| 2001 | 38449 | 15024 | 137 | 115 | 53 | 60 |
| 2002 | 39924 | 14619 | 129 | 111 | 40 | 42 |
| 2003 | 40894 | 14340 | 136 | 106 | 64 | 49 |
| 2004 | 44327 | 15043 | 130 | 126 | 48 | 54 |
| 2005 | 45516 | 15490 | 138 | 148 | 64 | 74 |
| 2006 | 48063 | 16309 | 135 | 133 | 58 | 69 |
| 2007 | 48373 | 16130 | 120 | 102 | 66 | 55 |
| 2008 | 47999 | 14946 | 134 | 93 | 53 | 53 |
| 2009 | 43449 | 17137 | 128 | 137 | 66 | 79 |
| 2010 | 44050 | 14155 | 123 | 106 | 61 | 56 |

**Table A4. Percent dead during follow-up within subgroups defined by GPA, graduation year, and country of birth or sex.**

|  | ***Native-born*** | | ***Foreign-born*** | |
| --- | --- | --- | --- | --- |
|  | *Medium/high GPA* | *Low GPA* | *Medium/high GPA* | *Low GPA* |
| ***All-cause mortality*** |  |  |  |  |
| *Graduation year* |  |  |  |  |
| 1990-1997 | 0.244 % | 0.610 % | 0.351 % | 0.585 % |
| 1998-2004 | 0.230 % | 0.652 % | 0.225 % | 0.491 % |
| 2005-2010 | 0.199 % | 0.638 % | 0.201 % | 0.383 % |
| **Deaths of despair** |  |  |  |  |
| *Graduation year* |  |  |  |  |
| 1990-1997 | 0.069 % | 0.218 % | 0.093 % | 0.213 % |
| 1998-2004 | 0.083 % | 0.287 % | 0.085 % | 0.184 % |
| 2005-2010 | 0.089 % | 0.354 % | 0.099 % | 0.156 % |
|  | ***Girls*** | | ***Boys*** | |
|  | *Medium/high GPA* | *Low GPA* | *Medium/high GPA* | *Low GPA* |
| ***All-cause mortality*** |  |  |  |  |
| *Graduation year* |  |  |  |  |
| 1990-1997 | 0.154 % | 0.320 % | 0.357 % | 0.764 % |
| 1998-2004 | 0.138 % | 0.363 % | 0.329 % | 0.794 % |
| 2005-2010 | 0.122 % | 0.358 % | 0.280 % | 0.764 % |
| **Deaths of despair** |  |  |  |  |
| *Graduation year* |  |  |  |  |
| 1990-1997 | 0.041 % | 0.112 % | 0.104 % | 0.274 % |
| 1998-2004 | 0.054 % | 0.163 % | 0.114 % % | 0.340 % |
| 2005-2010 | 0.048 % | 0.202 % | 0.133 % | 0.410 % |

**Table A5. Proportion with medium/high and low GPA within subgroups defined by graduation year and country of birth or sex.**

|  | *Medium/high GPA* | | *Low GPA* | |
| --- | --- | --- | --- | --- |
|  | *Native-born* | *Foreign-born* | *Native-born* | *Foreign-born* |
| *Graduation year* |  |  |  |  |
| 1990-1997 | 0.795 | 0.673 | 0.205 | 0.327 |
| 1998-2004 | 0.787 | 0.651 | 0.213 | 0.349 |
| 2005-2010 | 0.800 | 0.620 | 0.200 | 0.380 |
|  |  |  |  |  |
|  | *Girls* | *Boys* | *Girls* | *Boys* |
| *Graduation year* |  |  |  |  |
| 1990-1997 | 0.847 | 0.729 | 0.153 | 0.271 |
| 1998-2004 | 0.823 | 0.729 | 0.177 | 0.271 |
| 2005-2010 | 0.830 | 0.747 | 0.170 | 0.253 |

**Supplementary file B. Log-log plots**

Figure B1-B6 show log-log plots for all independent variables used in the analyses: GPA, graduation year, and the combination of (interaction between) graduation year and GPA. The plots are also shown for both outcomes: all-cause mortality and deaths of despair. There are no exact ways of determining whether log-log plots indicate support or violations of the proportional hazards assumption, but the lines for youths with low and medium/high GPA should run approximately parallel if the assumption is satisfied. The lines appear to be parallel for both all-cause mortality (Figures B1-B3) and deaths of despair (Figures B4-B6), supporting the proportional hazards assumption.

**Figure B1. Log-log plots, by GPA. Outcome = All-cause mortality**

**Figure B2. Log-log plots, by graduation year. Outcome = All-cause mortality**

**Figure B3. Log-log plots, by graduation year and GPA. Outcome = All-cause mortality**

**Figure B4. Log-log plots, by GPA. Outcome = Deaths of despair**

**Figure B5. Log-log plots, by graduation year. Outcome = Deaths of despair**

**Figure B6. Log-log plots, by graduation year and GPA. Outcome = Deaths of despair**

**Supplementary file C. Alternative operationalizations of deaths of despair**

Supplementary file C shows results for alternative ways of operationalizing deaths of despair. First, a broader indicator of deaths of despair was used, with other events of undetermined intent (ICD10: Y16–Y34; ICD9: E980–989) as well as deaths due to mental, behavioral and neurodevelopmental disorders (ICD10: F00–F99; ICD9: 290–319) included. The former category was included to account for possible misclassification of suicides (Allebeck et al., 1991; Gunnell et al., 2013; Spark et al., 2022).

Second, due to criticism that the components of deaths of despair are to diverse to be grouped into one category (Tilstra, 2023), two of the main components (suicide and overdose, respectively) were analyzed separately, using the definitions applied in the main manuscript. Thus, suicides (n= 1 904) were measured as follows: ICD10: X60–X84, Y87.0; ICD9: E950–E959. Overdoses (n=839) were measured as follows: X40–X45, Y10–Y15, Y45, Y47, Y49; ICD9: E850–E855, E858, E860, E935, E937, E939, E980.0–E980.5. The number of deaths from alcohol-related liver disease were too few (n=4) for a separate analysis to be feasible.

The results in Table C1, using a broader indicator of deaths of despair, are very similar to the main results. Tables C2 and C3 show that the association between low achievement and, respectively, suicide and overdoses did not change across graduation cohorts. The interaction terms between GPA and the 2005-2010 graduation cohort with suicide as the outcome were above 1 in all groups save for foreign-born youth, providing suggestive evidence of stronger associations in later graduation cohorts. However, the interactions were not statistically significant, possibly due to the smaller number of suicides compared to deaths of despair overall. The interaction terms with overdoses as the outcome were below 1 in all groups save for girls, providing suggestive evidence of weaker associations in later graduation cohorts. Again, possibly due to the smaller number of overdoses compared to deaths of despair overall, the interactions were not statistically significant.

It should be stressed, however, that the interaction terms measure relative differences. The main effects of graduation years show that overdoses but not suicides increased significantly for medium/high-achieving youths in the full sample and, in the stratified analyses, in native-born youths and boys. Likewise, the main effects of low GPA shows that, in the 1990-1997 cohort, educational inequalities in overdoses were larger than for suicides.

If overdoses increased in both low-and medium/high-achieving youths, but suicides were mostly stable in both groups, overdoses must logically have comprised a larger share of overall deaths of despair in later graduation cohorts. Likewise, if educational inequalities were larger for overdoses than for suicides, and these educational inequalities were stable over time in relative terms for both causes of death, overdoses must have accounted for a greater share of the educational inequalities. Moreover, if overdoses comprised a larger share of overall deaths of despair in later graduation cohorts, and educational inequalities were larger for overdoses, relative educational inequalities in DoD as a whole can increase *even though* relative inequalities in both suicides and overdoses were stable.

Figures C1-C3 illustrate these effects graphically. They are equivalent to Figures 1-3 in the main manuscript, but with suicides and overdoses instead of ACM and DoD.

**Figure C1. Proportion in % of youths that died during follow-up from suicide or drug overdose by GPA, three-year moving averages.**

**Figure C2. Proportion in % of youths that died during follow-up from suicide or drug overdose by GPA and country of birth, three-year moving averages.**

**Figure C3. Proportion in % of youths that died during follow-up from suicide or drug overdose by GPA and sex, three-year moving averages.**

**Table C1. Discrete time proportional hazard and competing risk models with deaths of despair or all other causes of death as outcomes.**

|  | *All* | *Native-born* | *Foreign-born* | *Girls* | *Boys* |
| --- | --- | --- | --- | --- | --- |
| *GPA (ref: medium/high GPA)* |  |  |  |  |  |
| Low GPA | 3.219*** | 3.266*** | 2.459*** | 2.862*** | 2.766*** |
|  | [2.833,3.659] | [2.853,3.739] | [1.645,3.673] | [2.177,3.761] | [2.388,3.203] |
| *Graduation year (ref: 1990-1997)* |  |  |  |  |  |
| 1998-2004 | 1.105 | 1.140* | 0.740 | 1.227 | 1.044 |
|  | [0.976,1.250] | [1.002,1.297] | [0.479,1.142] | [0.983,1.530] | [0.899,1.212] |
| 2005-2010 | 1.187** | 1.215** | 0.894 | 1.119 | 1.198* |
|  | [1.051,1.340] | [1.071,1.379] | [0.571,1.398] | [0.892,1.402] | [1.038,1.383] |
| *Graduation year X GPA* |  |  |  |  |  |
| 1998-2004 X Low GPA | 1.030 | 1.064 | 0.944 | 1.094 | 1.076 |
|  | [0.861,1.232] | [0.882,1.285] | [0.525,1.698] | [0.760,1.575] | [0.874,1.325] |
| 2005-2010 X Low GPA | 1.150 | 1.223* | 0.669 | 1.497* | 1.117 |
|  | [0.965,1.369] | [1.019,1.468] | [0.359,1.247] | [1.045,2.146] | [0.913,1.367] |
| N (individuals) | 2252938 | 2077701 | 175181 | 1099918 | 1152986 |
| N (observations) | 17791620 | 16437959 | 1353271 | 8666670 | 9124703 |

Table reports hazard ratios, with 95% confidence intervals in brackets. * p<0.05, ** p<0.01, *** p<0.001. Abbreviations: ref = reference category; GPA = Grade point average. Only results for deaths of despair shown in the table.

**Table C2. Cause-specific discrete time proportional hazard models with suicides vs. all other causes of death as outcomes.**

|  | *All* | *Native-born* | *Foreign-born* | *Girls* | *Boys* |
| --- | --- | --- | --- | --- | --- |
| *GPA (ref: medium/high GPA)* |  |  |  |  |  |
| Low GPA | 2.324*** | 2.407*** | 1.526 | 2.394*** | 1.933*** |
|  | [1.980,2.728] | [2.036,2.846] | [0.880,2.645] | [1.724,3.325] | [1.607,2.325] |
| *Graduation year (ref: 1990-1997)* |  |  |  |  |  |
| 1998-2004 | 1.116 | 1.135 | 0.886 | 1.263 | 1.044 |
|  | [0.970,1.285] | [0.980,1.314] | [0.536,1.463] | [0.986,1.619] | [0.880,1.238] |
| 2005-2010 | 1.042 | 1.046 | 1.002 | 1.116 | 0.996 |
|  | [0.903,1.202] | [0.902,1.213] | [0.593,1.695] | [0.864,1.442] | [0.839,1.183] |
| *Graduation year X GPA* |  |  |  |  |  |
| 1998-2004 X Low GPA | 0.923 | 0.902 | 1.270 | 0.930 | 0.971 |
|  | [0.736,1.158] | [0.710,1.145] | [0.607,2.661] | [0.596,1.452] | [0.744,1.266] |
| 2005-2010 X Low GPA | 1.189 | 1.236 | 0.830 | 1.408 | 1.180 |
|  | [0.950,1.487] | [0.979,1.560] | [0.372,1.850] | [0.912,2.173] | [0.907,1.536] |
| N (individuals) | 2252938 | 2077701 | 175181 | 1099918 | 1152986 |
| N (observations) | 17791651 | 16437983 | 1353278 | 8666687 | 9124717 |

Table reports hazard ratios, with 95% confidence intervals in brackets. * p<0.05, ** p<0.01, *** p<0.001. Abbreviations: ref = reference category; GPA = Grade point average. Only results for suicides are shown in the table.

**Table C3. Cause-specific discrete time proportional hazard models with overdoses vs. all other causes of death as outcomes.**

|  | *All* | *Native-born* | *Foreign-born* | *Girls* | *Boys* |
| --- | --- | --- | --- | --- | --- |
| *GPA (ref: medium/high GPA)* |  |  |  |  |  |
| Low GPA | 8.104*** | 8.134*** | 6.665*** | 5.192*** | 7.258*** |
|  | [5.924,11.09] | [5.846,11.32] | [2.441,18.19] | [2.567,10.50] | [5.070,10.39] |
| *Graduation year (ref: 1990-1997)* |  |  |  |  |  |
| 1998-2004 | 1.544* | 1.593* | 1.027 | 1.765 | 1.445 |
|  | [1.098,2.170] | [1.117,2.274] | [0.313,3.364] | [0.942,3.306] | [0.963,2.169] |
| 2005-2010 | 2.707*** | 2.832*** | 1.386 | 1.619 | 3.072*** |
|  | [1.989,3.683] | [2.056,3.901] | [0.423,4.542] | [0.855,3.064] | [2.153,4.385] |
| *Graduation year X GPA* |  |  |  |  |  |
| 1998-2004 X Low GPA | 1.098 | 1.202 | 0.517 | 1.401 | 1.135 |
|  | [0.731,1.650] | [0.785,1.841] | [0.126,2.124] | [0.590,3.328] | [0.708,1.820] |
| 2005-2010 X Low GPA | 0.763 | 0.816 | 0.453 | 1.671 | 0.672 |
|  | [0.523,1.112] | [0.551,1.210] | [0.110,1.863] | [0.700,3.988] | [0.439,1.028] |
| N (individuals) | 2252938 | 2077701 | 175181 | 1099918 | 1152986 |
| N (observations) | 17791709 | 16438032 | 1353287 | 8666708 | 9124754 |

Table reports hazard ratios, with 95% confidence intervals in brackets. * p<0.05, ** p<0.01, *** p<0.001. Abbreviations: ref = reference category; GPA = Grade point average. Only results for overdoses are shown in the table.

**Supplementary file D. Alternative operationalizations of achievement**

Supplementary file D explores alternative ways of operationalizing achievement. Tables D1 and D2 show results from logistic regression models with ACM and DoD as the outcomes and with GPA percentiles measured as a continuous variable (with a range of 1-100) as the focal independent variable. With a continuous measure of GPA, and no polynomial terms, the models assume that the log odds of death are a linear function of the GPA percentile scores. The negative linear association between achievement and all-cause mortality became stronger (i.e., more negative) for later graduation cohorts in the full sample, in native-born youths and in both girls and boys (Table D1), while the negative linear association between achievement and deaths of despair became stronger in the full sample and in native-born youths, but weaker (i.e., less negative) in foreign-born youth (Table D2).

The composition of the group with low GPA may have changed across graduation cohorts due to immigration, with relatively more immigrant youths among those with low grades in more recent cohorts. If so, changes in the association between GPA and internalizing disorders across cohorts may partly be due to compositional changes. We stress that, since the aim of the present study is descriptive, this would not be a source of bias, but it would influence the interpretation of the results. Table D3 shows results of analyses in which the low GPA category was calculated separately for native-born and immigrant youths. That is, in the analyses of native born youths, low GPA indicate the 20% of native born youths with the lowest GPA, and conversely for the analyses of foreign-born youths. The results for native-born youths are similar to the main results, and show a stronger association between low achievement and both all-cause mortality and deaths of despair in later graduation cohorts. In contrast to the main results, the results for foreign-born youths however show that the association between low achievement and both all-cause mortality and deaths of despair became significantly weaker in later graduation cohorts when using subgroup specific cutoffs for academic achievement.

Tables D4 and D5 show results with stricter definition of low achievement, with only the 1^st^ to the 10^th^ GPA percentiles indicating low GPA. The results are again similar to the main results, and show that the association between low achievement and all-cause mortality became stronger in the full sample, in native-born youth, and in both girls and boys. The association between low achievement and deaths of despair became stronger in native-born youth and girls.

Tables D6 and D7 show results with broader definition of low achievement, with the 1^st^ to the 30^th^ GPA percentiles indicating low GPA. The results are again similar to the main results, and show that the association between low achievement and all-cause mortality became stronger in the full sample, in native-born youth, and in girls, but not in boys. The association between low achievement and deaths of despair became stronger in native-born youth and girls, and weaker in foreign-born youths.

**Table D1. Discrete time proportional hazard models with all-cause mortality as the outcome**

|  | *All* | *Native-born* | *Foreign-born* | *Girls* | *Boys* |
| --- | --- | --- | --- | --- | --- |
| *GPA* |  |  |  |  |  |
| GPA percentile | 0.982*** | 0.982*** | 0.987*** | 0.987*** | 0.984*** |
|  | [0.981,0.984] | [0.981,0.984] | [0.982,0.992] | [0.985,0.990] | [0.982,0.986] |
| *Graduation year (ref: 1990-1997)* |  |  |  |  |  |
| 1998-2004 | 1.045 | 1.087 | 0.822 | 1.071 | 1.062 |
|  | [0.953,1.147] | [0.985,1.199] | [0.619,1.093] | [0.881,1.303] | [0.955,1.182] |
| 2005-2010 | 0.984 | 1.054 | 0.550*** | 1.099 | 0.982 |
|  | [0.895,1.082] | [0.954,1.165] | [0.400,0.757] | [0.902,1.338] | [0.879,1.096] |
| *Graduation year X GPA* |  |  |  |  |  |
| 1998-2004 X GPA percentile | 0.999 | 0.998 | 0.996 | 0.998 | 0.998 |
|  | [0.996,1.001] | [0.996,1.001] | [0.989,1.004] | [0.995,1.002] | [0.995,1.001] |
| 2005-2010 X GPA percentile | 0.997** | 0.996*** | 1.004 | 0.995* | 0.997* |
|  | [0.995,0.999] | [0.994,0.998] | [0.996,1.012] | [0.992,0.999] | [0.994,0.999] |
| N (individuals) | 2252938 | 2077701 | 175181 | 1099918 | 1152986 |
| N (observations) | 17791418 | 16437773 | 1353255 | 8666587 | 9124584 |

Table reports hazard ratios, with 95% confidence intervals in brackets. * p<0.05, ** p<0.01, *** p<0.001. Abbreviations: ref = reference category; GPA = Grade point average.

**Table D2. Cause-specific discrete time proportional hazard models with deaths of despair vs. all other causes of death as outcomes.**

|  | *All* | *Native-born* | *Foreign-born* | *Girls* | *Boys* |
| --- | --- | --- | --- | --- | --- |
| *GPA* |  |  |  |  |  |
| GPA percentile | 0.979*** | 0.979*** | 0.981*** | 0.983*** | 0.981*** |
|  | [0.976,0.982] | [0.976,0.982] | [0.971,0.990] | [0.978,0.988] | [0.978,0.984] |
| *Graduation year (ref: 1990-1997)* |  |  |  |  |  |
| 1998-2004 | 1.308*** | 1.425*** | 0.672 | 1.520* | 1.308** |
|  | [1.122,1.524] | [1.213,1.675] | [0.412,1.097] | [1.099,2.100] | [1.097,1.560] |
| 2005-2010 | 1.575*** | 1.752*** | 0.567* | 1.848*** | 1.554*** |
|  | [1.357,1.827] | [1.499,2.048] | [0.337,0.953] | [1.346,2.536] | [1.310,1.843] |
| *Graduation year X GPA* |  |  |  |  |  |
| 1998-2004 X GPA percentile | 0.999 | 0.997 | 1.010 | 0.998 | 0.997 |
|  | [0.995,1.002] | [0.993,1.001] | [0.997,1.023] | [0.992,1.005] | [0.992,1.002] |
| 2005-2010 X GPA percentile | 0.996* | 0.994** | 1.015* | 0.994 | 0.996 |
|  | [0.992,0.999] | [0.990,0.998] | [1.002,1.029] | [0.987,1.000] | [0.992,1.001] |
| N (individuals) | 2252938 | 2077701 | 175181 | 1099918 | 1152986 |
| N (observations) | 17791418 | 16437773 | 1353255 | 8666587 | 9124584 |

Table reports hazard ratios, with 95% confidence intervals in brackets. * p<0.05, ** p<0.01, *** p<0.001. Abbreviations: ref = reference category; GPA = Grade point average. Only results for deaths of despair shown in the table.

**Table D3. Discrete time proportional hazard models with all-cause mortality as the outcome, and cause-specific discrete time proportional hazard models with deaths of despair vs. all other causes of death as outcomes.**

|  | *All-cause mortality* | | *Deaths of despair* | |
| --- | --- | --- | --- | --- |
|  | *Native-born* | *Foreign-born* | *Native-born* | *Foreign-born* |
| *GPA (ref: medium/high GPA)* |  |  |  |  |
| Low GPA | 2.517*** | 2.004*** | 3.189*** | 2.770*** |
|  | [2.318,2.733] | [1.541,2.607] | [2.760,3.685] | [1.751,4.380] |
| *Graduation year (ref: 1990-1997)* |  |  |  |  |
| 1998-2004 | 0.952 | 0.735** | 1.213** | 1.099 |
|  | [0.882,1.028] | [0.584,0.926] | [1.059,1.389] | [0.735,1.644] |
| 2005-2010 | 0.802*** | 0.752* | 1.255*** | 1.335 |
|  | [0.739,0.869] | [0.588,0.962] | [1.097,1.437] | [0.888,2.006] |
| *Graduation year X GPA* |  |  |  |  |
| 1998-2004 X Low GPA | 1.124 | 1.064 | 1.082 | 0.607 |
|  | [0.998,1.266] | [0.724,1.564] | [0.888,1.320] | [0.313,1.178] |
| 2005-2010 X Low GPA | 1.238*** | 0.559* | 1.217* | 0.271** |
|  | [1.097,1.398] | [0.347,0.901] | [1.004,1.476] | [0.120,0.614] |
| N (individuals) | 2077701 | 175181 | 2077701 | 175181 |
| N (observations) | 16437773 | 1353255 | 16437980 | 1353278 |

Table reports hazard ratios, with 95% confidence intervals in brackets. * p<0.05, ** p<0.01, *** p<0.001. Abbreviations: ref = reference category; GPA = Grade point average.

**Table D4. Discrete time proportional hazard models with all-cause mortality as the outcome.**

|  | *All* | *Native-born* | *Foreign-born* | *Girls* | *Boys* |
| --- | --- | --- | --- | --- | --- |
| *GPA* |  |  |  |  |  |
| Low GPA (bottom 10%) | 2.605*** | 2.681*** | 1.843*** | 2.122*** | 2.310*** |
|  | [2.384,2.846] | [2.442,2.943] | [1.394,2.435] | [1.726,2.609] | [2.092,2.551] |
| *Graduation year (ref: 1990-1997)* |  |  |  |  |  |
| 1998-2004 | 0.970 | 0.997 | 0.689** | 0.945 | 0.969 |
|  | [0.909,1.035] | [0.932,1.067] | [0.550,0.864] | [0.839,1.063] | [0.897,1.046] |
| 2005-2010 | 0.838*** | 0.856*** | 0.650*** | 0.798*** | 0.843*** |
|  | [0.784,0.897] | [0.799,0.918] | [0.505,0.836] | [0.705,0.904] | [0.778,0.913] |
| *Graduation year X GPA* |  |  |  |  |  |
| 1998-2004 X (bottom 10%) | 1.117 | 1.149* | 1.166 | 1.237 | 1.177* |
|  | [0.982,1.271] | [1.002,1.318] | [0.785,1.731] | [0.930,1.646] | [1.017,1.361] |
| 2005-2010 X (bottom 10%) | 1.267*** | 1.375*** | 0.791 | 1.691*** | 1.280** |
|  | [1.111,1.444] | [1.199,1.578] | [0.506,1.238] | [1.279,2.236] | [1.101,1.487] |
| N (individuals) | 2252938 | 2077701 | 175181 | 1099918 | 1152986 |
| N (observations) | 17791418 | 16437773 | 1353255 | 8666587 | 9124584 |

Table reports hazard ratios, with 95% confidence intervals in brackets. * p<0.05, ** p<0.01, *** p<0.001. Abbreviations: ref = reference category; GPA = Grade point average.

**Table D5. Cause-specific discrete time proportional hazard models with deaths of despair vs. all other causes of death as outcomes.**

|  | *All* | *Native-born* | *Foreign-born* | *Girls* | *Boys* |
| --- | --- | --- | --- | --- | --- |
| *GPA* |  |  |  |  |  |
| Low GPA (bottom 10%) | 3.388*** | 3.508*** | 2.271*** | 2.687*** | 2.982*** |
|  | [2.918,3.933] | [2.998,4.104] | [1.403,3.677] | [1.881,3.838] | [2.525,3.523] |
| *Graduation year (ref: 1990-1997)* |  |  |  |  |  |
| 1998-2004 | 1.214*** | 1.234*** | 0.972 | 1.302* | 1.164* |
|  | [1.084,1.360] | [1.096,1.390] | [0.662,1.426] | [1.057,1.603] | [1.017,1.332] |
| 2005-2010 | 1.346*** | 1.372*** | 1.072 | 1.190 | 1.386*** |
|  | [1.205,1.503] | [1.223,1.540] | [0.715,1.607] | [0.962,1.472] | [1.217,1.577] |
| *Graduation year X GPA* |  |  |  |  |  |
| 1998-2004 X (bottom 10%) | 1.134 | 1.232 | 0.731 | 1.401 | 1.182 |
|  | [0.924,1.392] | [0.994,1.528] | [0.371,1.441] | [0.891,2.202] | [0.936,1.493] |
| 2005-2010 X (bottom 10%) | 1.164 | 1.294* | 0.514 | 1.946** | 1.103 |
|  | [0.953,1.422] | [1.050,1.594] | [0.248,1.065] | [1.255,3.020] | [0.878,1.385] |
| N (individuals) | 2252938 | 2077701 | 175181 | 1099918 | 1152986 |
| N (observations) | 17791648 | 16437980 | 1353278 | 8666686 | 9124715 |

Table reports hazard ratios, with 95% confidence intervals in brackets. * p<0.05, ** p<0.01, *** p<0.001. Abbreviations: ref = reference category; GPA = Grade point average. Only results for deaths of despair shown in the table.

**Table D6. Discrete time proportional hazard models with all-cause mortality as the outcome.**

|  | *All* | *Native-born* | *Foreign-born* | *Girls* | *Boys* |
| --- | --- | --- | --- | --- | --- |
| *GPA* |  |  |  |  |  |
| Low GPA (bottom 30%) | 2.323*** | 2.329*** | 2.031*** | 1.906*** | 2.092*** |
|  | [2.151,2.509] | [2.149,2.524] | [1.561,2.642] | [1.637,2.219] | [1.910,2.291] |
| *Graduation year (ref: 1990-1997)* |  |  |  |  |  |
| 1998-2004 | 0.927 | 0.943 | 0.715* | 0.900 | 0.936 |
|  | [0.854,1.006] | [0.866,1.027] | [0.527,0.969] | [0.782,1.036] | [0.846,1.036] |
| 2005-2010 | 0.812*** | 0.818*** | 0.741 | 0.797** | 0.817*** |
|  | [0.746,0.885] | [0.749,0.894] | [0.532,1.032] | [0.689,0.923] | [0.735,0.908] |
| *Graduation year X GPA* |  |  |  |  |  |
| 1998-2004 X Low GPA (bottom 30%) | 1.119* | 1.142* | 1.070 | 1.255* | 1.089 |
|  | [1.000,1.252] | [1.016,1.285] | [0.729,1.569] | [1.008,1.562] | [0.953,1.243] |
| 2005-2010 X Low GPA (bottom 30%) | 1.139* | 1.191** | 0.744 | 1.313* | 1.104 |
|  | [1.014,1.279] | [1.056,1.344] | [0.486,1.139] | [1.049,1.642] | [0.962,1.267] |
| N (individuals) | 2252938 | 2077701 | 175181 | 1099918 | 1152986 |
| N (observations) | 17791418 | 16437773 | 1353255 | 8666587 | 9124584 |

Table reports hazard ratios, with 95% confidence intervals in brackets. * p<0.05, ** p<0.01, *** p<0.001. Abbreviations: ref = reference category; GPA = Grade point average.

**Table D7. Cause-specific discrete time proportional hazard models with deaths of despair vs. all other causes of death as outcomes.**

|  | *All* | *Native-born* | *Foreign-born* | *Girls* | *Boys* |
| --- | --- | --- | --- | --- | --- |
| *GPA* |  |  |  |  |  |
| Low GPA (bottom 30%) | 2.726*** | 2.683*** | 2.987*** | 2.383*** | 2.362*** |
|  | [2.373,3.131] | [2.321,3.101] | [1.802,4.952] | [1.804,3.149] | [2.009,2.777] |
| *Graduation year (ref: 1990-1997)* |  |  |  |  |  |
| 1998-2004 | 1.163* | 1.156 | 1.228 | 1.254 | 1.115 |
|  | [1.007,1.345] | [0.994,1.343] | [0.708,2.130] | [0.974,1.614] | [0.935,1.331] |
| 2005-2010 | 1.226** | 1.210* | 1.499 | 1.174 | 1.244* |
|  | [1.062,1.416] | [1.043,1.403] | [0.851,2.639] | [0.908,1.519] | [1.047,1.479] |
| *Graduation year X GPA* |  |  |  |  |  |
| 1998-2004 X Low GPA (bottom 30%) | 1.114 | 1.192 | 0.630 | 1.302 | 1.095 |
|  | [0.920,1.348] | [0.976,1.456] | [0.321,1.238] | [0.899,1.884] | [0.873,1.372] |
| 2005-2010 X Low GPA (bottom 30%) | 1.206 | 1.316** | 0.442* | 1.486* | 1.145 |
|  | [1.000,1.454] | [1.083,1.600] | [0.217,0.897] | [1.027,2.150] | [0.920,1.427] |
| N (individuals) | 2252938 | 2077701 | 175181 | 1099918 | 1152986 |
| N (observations) | 17791648 | 16437980 | 1353278 | 8666686 | 9124715 |

Table reports hazard ratios, with 95% confidence intervals in brackets. * p<0.05, ** p<0.01, *** p<0.001. Abbreviations: ref = reference category; GPA = Grade point average. Only results for deaths of despair shown in the table.

**Supplementary file E. Alternative operationalizations of graduation year**

Additional file E investigates alternative ways of measuring time, that is, graduation years or cohorts. Tables E1 and E2 shows results with graduation years entered as a continuous variable in the models, and with low GPA operationalized as in the main models (i.e., as the bottom 20%). Graduation years have been recoded and centered at 1990, meaning that 0 represents 1990, 1 represents 1991 and so on. Results show that the association between low achievement and all-cause mortality became linearly stronger for later graduation cohorts in the full sample, in native-born youth, and in both girls and boys, while the association between low achievement and deaths of despair only became linearly stronger in native-born youth and in girls.

Tables E3 and E4 shows results with both graduation years and GPA percentiles entered as continuous variables. The main effect of GPA thus shows the linear association between GPA and all-cause mortality or deaths of despair in the 1990 graduation cohort, and the interaction term show how this linear association changes for each successive graduation cohort, assuming a linear change across graduation cohorts.

Figure E1 shows the proportion in % of youths that died during follow-up from all causes or from despair by GPA and graduation year, and Figure E2 the equivalent but separately depending on country of birth and sex. They are similar to Figures 2-3 in the main manuscript, but without using 3-year moving averages for the proportions. The results are also qualitatively similar to those in Figures 2-3, but with more short-term fluctuations around the more long-term trends.

With all-cause mortality as the outcome, the interaction between graduation year and GPA is significant and negative in the full sample, in native-born youth, and both girls and boys, indicating a stronger negative association between GPA and all-cause mortality in later graduation cohorts. With deaths of despair as the outcome, the interaction between graduation year and GPA is significant and negative in native-born youth and in girls, indicating a stronger negative association between GPA and deaths of despair in later graduation cohorts, and significant and negative in foreign-born youth.

**Table E1. Discrete time proportional hazard models with all-cause mortality as the outcome**

|  | *All* | *Native-born* | *Foreign-born* | *Girls* | *Boys* |
| --- | --- | --- | --- | --- | --- |
| *GPA (ref: medium/high GPA)* |  |  |  |  |  |
| Low GPA | 2.303*** | 2.356*** | 1.640** | 1.899*** | 2.049*** |
|  | [2.101,2.525] | [2.140,2.594] | [1.200,2.240] | [1.568,2.299] | [1.843,2.279] |
| *Graduation year* |  |  |  |  |  |
| Graduation year | 0.984*** | 0.986*** | 0.956*** | 0.980*** | 0.985*** |
|  | [0.979,0.989] | [0.981,0.991] | [0.937,0.975] | [0.972,0.989] | [0.979,0.991] |
| *Graduation year X GPA* |  |  |  |  |  |
| Graduation year X Low GPA | 1.016*** | 1.018*** | 1.017 | 1.028*** | 1.016*** |
|  | [1.008,1.024] | [1.009,1.026] | [0.989,1.046] | [1.012,1.044] | [1.007,1.025] |
| N (individuals) | 2252938 | 2077701 | 175181 | 1099918 | 1152986 |
| N (observations) | 17791418 | 16437773 | 1353255 | 8666587 | 9124584 |

Table reports hazard ratios, with 95% confidence intervals in brackets. * p<0.05, ** p<0.01, *** p<0.001. Abbreviations: ref = reference category; GPA = Grade point average.

**Table E2. Cause-specific discrete time proportional hazard models with deaths of despair vs. all other causes of death as outcomes.**

|  | *All* | *Native-born* | *Foreign-born* | *Girls* | *Boys* |
| --- | --- | --- | --- | --- | --- |
| *GPA (ref: medium/high GPA)* |  |  |  |  |  |
| Low GPA | 2.981*** | 3.013*** | 2.564*** | 2.371*** | 2.709*** |
|  | [2.545,3.492] | [2.553,3.556] | [1.488,4.417] | [1.720,3.270] | [2.253,3.258] |
| *Graduation year* |  |  |  |  |  |
| Graduation year | 1.016*** | 1.018*** | 0.996 | 1.004 | 1.021*** |
|  | [1.008,1.025] | [1.009,1.027] | [0.963,1.030] | [0.989,1.020] | [1.010,1.031] |
| *Graduation year X GPA* |  |  |  |  |  |
| Graduation year X Low GPA | 1.011 | 1.015* | 0.978 | 1.031* | 1.007 |
|  | [0.998,1.023] | [1.002,1.028] | [0.934,1.024] | [1.006,1.057] | [0.992,1.021] |
| N (individuals) | 2252938 | 2077701 | 175181 | 1099918 | 1152986 |
| N (observations) | 17791648 | 16437980 | 1353278 | 8666686 | 9124715 |

Table reports hazard ratios, with 95% confidence intervals in brackets. * p<0.05, ** p<0.01, *** p<0.001. Abbreviations: ref = reference category; GPA = Grade point average. Only results for deaths of despair shown in the table.

**Table E3. Discrete time proportional hazard models with all-cause mortality as the outcome.**

|  | *All* | *Native-born* | *Foreign-born* | *Girls* | *Boys* |
| --- | --- | --- | --- | --- | --- |
| *GPA* |  |  |  |  |  |
| GPA percentile | 0.9833*** | 0.9832*** | 0.9869*** | 0.9888*** | 0.9845*** |
|  | [0.9817,0.9850] | [0.9815,0.9850] | [0.9808,0.9931] | [0.9858,0.9918] | [0.9824,0.9866] |
| *Graduation year* |  |  |  |  |  |
| Graduation year | 0.9992 | 1.0034 | 0.9649*** | 1.0053 | 0.9991 |
|  | [0.9929,1.0055] | [0.9968,1.0100] | [0.9446,0.9856] | [0.9922,1.0186] | [0.9919,1.0064] |
| *Graduation year X GPA* |  |  |  |  |  |
| Graduation year X GPA percentile | 0.9998** | 0.9997*** | 1.0000 | 0.9997** | 0.9998* |
|  | [0.9996,0.9999] | [0.9996,0.9999] | [0.9994,1.0006] | [0.9994,0.9999] | [0.9996,1.0000] |
| N (individuals) | 2252938 | 2077701 | 175181 | 1099918 | 1152986 |
| N (observations) | 17791418 | 16437773 | 1353255 | 8666587 | 9124584 |

Table reports hazard ratios, with 95% confidence intervals in brackets. * p<0.05, ** p<0.01, *** p<0.001. Abbreviations: ref = reference category; GPA = Grade point average.

**Table E4. Cause-specific discrete time proportional hazard models with deaths of despair vs. all other causes of death as outcomes.**

|  | *All* | *Native-born* | *Foreign-born* | *Girls* | *Boys* |
| --- | --- | --- | --- | --- | --- |
| *GPA* |  |  |  |  |  |
| GPA percentile | 0.9797*** | 0.9799*** | 0.9788*** | 0.9853*** | 0.9805*** |
|  | [0.9766,0.9827] | [0.9767,0.9830] | [0.9678,0.9899] | [0.9800,0.9908] | [0.9767,0.9844] |
| *Graduation year* |  |  |  |  |  |
| Graduation year | 1.0302*** | 1.0378*** | 0.9579* | 1.0369*** | 1.0296*** |
|  | [1.0200,1.0405] | [1.0270,1.0486] | [0.9245,0.9925] | [1.0156,1.0586] | [1.0178,1.0415] |
| *Graduation year X GPA* |  |  |  |  |  |
| Graduation year X GPA percentile | 0.9998 | 0.9996** | 1.0010* | 0.9995* | 0.9998 |
|  | [0.9995,1.0000] | [0.9994,0.9999] | [1.0001,1.0019] | [0.9991,1.0000] | [0.9995,1.0001] |
| N (individuals) | 2252938 | 2077701 | 175181 | 1099918 | 1152986 |
| N (observations) | 17791648 | 16437980 | 1353278 | 8666686 | 9124715 |

Table reports hazard ratios, with 95% confidence intervals in brackets. * p<0.05, ** p<0.01, *** p<0.001. Abbreviations: ref = reference category; GPA = Grade point average. Only results for deaths of despair shown in the table.

**Fig.E1. Proportion in % of youths that died during follow-up from all causes or from despair by GPA, for each graduation year.**

Abbreviations: ACM= all-cause mortality; DoD = Deaths of despair; GPA = Grade point average

**Fig.E2. Proportion in % of youths that died during follow-up from all causes or from despair by GPA and country of birth or sex, for each graduation year.**

Abbreviations: ACM= all-cause mortality; DoD = Deaths of despair; GPA = Grade point average

**Supplementary file F. Subdistribution hazard models**

A key assumption underlying cause-specific hazard models is that the competing events are independent of each other (Lau et al., 2009). This is similar to the “independent censoring” assumption – stating that censoring is unrelated to the likelihood of the event – underlying all survival analysis techniques. Subdistribution hazard models (Fine & Gray, 1999) relaxes this assumption by keeping units experiencing the competing event in the risk set for the estimation of the hazard of the focal event. Cause-specific hazard models are generally regarded as more appropriate for analyzing the aetiology of diseases or causes of death, while subdistribution hazard models are more appropriate for prognostic purposes since it is better suited for estimating the cumulative incidence function (Austin & Fine, 2017). We argue that the aim of this study is more aligned with the advantages of the cause-specific hazard models. Nonetheless, the assumption of independence between competing events is unlikely to hold exactly, and when, as in this case, the association between the focal exposure (i.e., the interaction between low achievement and graduation cohorts) and both the focal and the competing events are above 1, subdistribution hazards will be smaller than cause-specific hazards (Lau et al., 2009). Subdistribution hazard models require continuous time-to-event data, which were not available for this study, but methods for continuous data can be used as an approximation for discrete data (Schmid & Berger, 2021). Supplementary file G shows that results using subdistribution hazard models (Fine & Gray, 1999) on the discrete data produced almost identical results as the cause-specific hazard models.

The assumption of independence between competing events is unlikely to hold exactly, and when, as in this case, the association between the focal exposure (i.e., the interaction between low achievement and graduation cohorts) and both the focal and the competing events are above 1, subdistribution hazards will be smaller than cause-specific hazards (Lau et al., 2009). Subdistribution hazard models require continuous time-to-event data, which were not available for this study, but methods for continuous data can be used as an approximation for discrete data (Schmid & Berger, 2021). Table F1 shows that results using subdistribution hazard models on the discrete data produced almost identical results as the cause-specific hazard models.

**Table F1. Subdistribution hazard models with deaths of despair vs. all other causes of death as outcomes.**

|  | *All* | *Native-born* | *Foreign-born* | *Girls* | *Boys* |
| --- | --- | --- | --- | --- | --- |
| *GPA (ref: medium/high GPA)* |  |  |  |  |  |
| Low GPA | 3.069*** | 3.130*** | 2.317*** | 2.769*** | 2.631*** |
|  | [2.674,3.523] | [2.708,3.618] | [1.468,3.657] | [2.065,3.712] | [2.246,3.082] |
| *Graduation year (ref: 1990-1997)* |  |  |  |  |  |
| 1998-2004 | 1.174* | 1.196** | 0.906 | 1.332* | 1.097 |
|  | [1.031,1.336] | [1.045,1.369] | [0.571,1.438] | [1.058,1.677] | [0.937,1.284] |
| 2005-2010 | 1.261*** | 1.278*** | 1.058 | 1.185 | 1.274** |
|  | [1.110,1.431] | [1.120,1.459] | [0.655,1.708] | [0.935,1.501] | [1.095,1.481] |
| *Graduation year X GPA* |  |  |  |  |  |
| 1998-2004 X Low GPA | 1.067 | 1.101 | 0.941 | 1.088 | 1.131 |
|  | [0.883,1.289] | [0.903,1.343] | [0.500,1.773] | [0.740,1.598] | [0.907,1.410] |
| 2005-2010 X Low GPA | 1.202 | 1.274* | 0.688 | 1.521* | 1.176 |
|  | [0.999,1.446] | [1.051,1.545] | [0.351,1.348] | [1.040,2.225] | [0.951,1.456] |
| N (individuals) | 2252938 | 2077701 | 175181 | 1099918 | 1152986 |
| N (observations) | 17789010 | 16436465 | 1352158 | 8665374 | 9123390 |

Table reports subdistribution hazard ratios, with 95% confidence intervals in brackets. * p<0.05, ** p<0.01, *** p<0.001. Abbreviations: ref = reference category; GPA = Grade point average. Only results for deaths of despair shown in the table.

**Supplementary file G. Additive interactions.**

Additive interactions were investigated in supplementary file G. Let the first subscript represent achievement (0= medium/high GPA; 1=low GPA) and the second subscript graduation cohorts (0=1990-1997; 1=2005-2010; leaving out the 1998-2004 cohort for brevity). In a comparison of low and medium/high achieving youth in the 1990-1997 and 2005-2010 graduation cohorts, RR_10_ is then the risk ratio for low achieving youth in the 1990-1997 cohort, RR_01_ the risk ratio medium/high achieving youth in the 2005-2010 cohort, and RR_11_ the risk ratio for low achieving youth in the 2005-2010 cohort, with all groups compared with medium/high achieving youth in the 1990-1997 cohort (RR_00_).

Multiplicative interaction measures if the joint effect of two exposures (e.g., low achievement and later graduation years) differs from the product of their individual effects, as measured on a relative scale. Multiplicative interaction is less sensitive to differences in baseline risks. Multiplicative interaction was measured as RR_11_ / (RR_10_X RR_01_), which is also what was presented in the main manuscript.

Additive interaction was investigated in two ways. Firstly, by the relative excess risk due to interaction (RERI), which measures if the combined “effect” of two exposure (e.g., low achievement and later graduation years) exceeds the sum of their individual effects, and is defined as RERI = RR_11_ - RR_10_ - RR_01_ + 1. A RERI larger than 0 indicates that there is an excess risk due to interaction. Secondly by the attributable proportion (AP), which measures how much of the total risk in the doubly exposed group (i.e., low achieving youth in the 2005-2010 graduation cohort) that can be accounted for by the relative excess risk due to interaction, and is defined as AP = (RR_11_ - RR_10_ - RR_01_ + 1) / RR_11_. An AP larger than 0 indicates that the excess risk due to interaction adds to the total risk in the doubly exposed group.

Additive interaction is typically regarded as being more relevant from a public health perspective since it measures how much the actual burden of a disease or cause of death differs depending on combination of exposures (e.g., graduation year and GPA or UPS completion) (VanderWeele & Knol, 2014).

For brevity, only results comparing the 1990-1997 to the 2005-2010 graduation cohorts is shown in the tables below. Table G1 shows that, with all-cause mortality as the outcome, the two measures of additive interaction were only significant and positive for native-born youths and girls.

With deaths of despair as the outcome (Table G2), however, both measures were significant and positive in the full samples as well as in all subgroups save for foreign-born youths. Specifically, the RERI of 1.321 in the full sample means that the relative risk of DoD for low-achieving youths in the 2005-2010 graduation cohort is 1.3 “units” (i.e., hazard ratios) greater than what would be expected from the “effects” of low GPA and the 2005-2010 graduation cohort in isolation. The AP of 0.283 means that these 1.3 “excess units” (i.e., the relative excess risk due to interaction) accounts for around 28% of the total risk of DoD for low-achieving youths in the 2005-2010 graduation cohort.

**Table G1. Relative excess risk due to interaction and attributable proportions due both low achievement and graduation year: All-cause mortality**

| Measure | *Full sample* | *Native-born* | *Foreign-born* | *Girls* | *Boys* |
| --- | --- | --- | --- | --- | --- |
| RR_00_  High/medium GPA 1990-1997 | 1 | 1 | 1 | 1 | 1 |
| RR_10_  Low GPA 1990-1997 | 2.434 | 2.502 | 1.686 | 2.075 | 2.149 |
| RR_01_  High/medium GPA 2005-2010 | 0.795 | 0.816 | 0.569 | 0.788 | 0.788 |
| RR_11_  Low GPA 2005-2010 | 2.424 | 2.617 | 1.096 | 2.322 | 2.151 |
| RERI | 0.196  [-0.027,0.419] | 0.299*  [0.053,0.544] | -0.159  [-0. 629,0. 310] | 0.459*  [0.022,0.897] | 0.214  [-0.010,0.441] |
| AP | 0.080  [-0.008,0.170] | 0.114*  [0.024,0.203] | -0.146  [-0.583,0.291] | 0.198*  [0.024,0.371] | 0.100  [-0.002,0.201] |

Notes: RERI = RR_11_ - RR_10_ - RR_01_ + 1. AP = (RR_11_ - RR_10_ - RR_01_ + 1) / RR_11_. 95% confidence intervals in parenthesis. Abbreviations: RR = Risk ratio. GPA = Grade point average. RERI = Relative excess risk due to interaction. AP = Attributable proportion.

**Table G2. Relative excess risk due to interaction and attributable proportions due both low achievement and graduation year: Deaths of despair**

| Measure | *Full sample* | *Native-born* | *Foreign-born* | *Girls* | *Boys* |
| --- | --- | --- | --- | --- | --- |
| RR_00_  High/medium GPA 1990-1997 | 1 | 1 | 1 | 1 | 1 |
| RR_10_  Low GPA 1990-1997 | 3.073 | 3.134 | 2.319 | 2.770 | 2.635 |
| RR_01_  High/medium GPA 2005-2010 | 1.260 | 1.278 | 1.057 | 1.185 | 1.273 |
| RR_11_  Low GPA 2005-2010 | 4.654 | 5.103 | 1.686 | 4.991 | 3.946 |
| RERI | 1.321***  [0.796,1.845] | 1.691***  [1.107,2.275] | -0.689  [-1.842,0.463] | 2.037***  [0.899,3.173] | 1.038***  [0.528,1.548] |
| AP | 0.283***  [0.187,0.380] | 0.331***  [0.237,0.425] | -0.408  [-1.128,0.310] | 0.408***  [0.231,0.585] | 0.263***  [0.149,0.376] |

Notes: RERI = RR_11_ - RR_10_ - RR_01_ + 1. AP = (RR_11_ - RR_10_ - RR_01_ + 1) / RR_11_. 95% confidence intervals in parenthesis. Abbreviations: RR = Risk ratio. GPA = Grade point average. RERI = Relative excess risk due to interaction. AP = Attributable proportion.

**Supplementary file H. Alternative measures of country of birth**

Supplementary file H shows results using a more fine-grained measure of country of birth, separating native-born youths with a Swedish background (two native-born parents), native-born youths with a Western background (at least one parent born in, but no parent born outside, Europe or North America), native-born youths with a non-Western background (at least one parent born outside Europe or North America), foreign-born youths with a Western background, and foreign-born youths with a non-Western background. It should be noted that the sample within some of these more fine-grained categories was fairly small, resulting in large confidence intervals. Thus, the specific point estimates should be taken with a pinch of salt.

The association between low achievement and all-cause mortality became stronger for more recent graduation cohorts in native-born youth with Swedish background and in foreign-born youth with Western background (Table H1), while the association between low achievement and deaths of despair only became stronger for more recent graduation cohorts in native-born youth with Swedish background (Table H2).

Only data on larger groups of countries, or continents, were available regarding the country of birth of the participants. Table H3 shows the number of observations and the percent of the sample for each country or region of birth of the participants and the participant’s parents, using the maximally detailed categorization available in the data. Table H4 shows the 10 most common countries of birth apart from Sweden in the population aged 15-24, as well as proportion of the total population in that age group that was born in that country. The numbers refer to the years 2000-2018, which is the only years with available data from Statistics Sweden (2024).

**Table H1. Discrete time proportional hazard models with all-cause mortality as the outcome.**

|  | *Native-born & Swedish background* | *Native-born & Western background* | *Native-born & non-Western background* | *Foreign-born & Western background* | *Foreign-born & non-Western background* |
| --- | --- | --- | --- | --- | --- |
| *GPA (ref: medium/high GPA)* |  |  |  |  |  |
| Low GPA | 2.463*** | 2.319*** | 4.143*** | 1.419 | 1.874*** |
|  | [2.245,2.703] | [1.910,2.814] | [2.436,7.045] | [0.934,2.157] | [1.356,2.590] |
| *Graduation year (ref: 1990-1997)* |  |  |  |  |  |
| 1998-2004 | 0.989 | 0.761** | 0.756 | 0.484*** | 0.762 |
|  | [0.910,1.076] | [0.619,0.935] | [0.456,1.252] | [0.317,0.739] | [0.546,1.065] |
| 2005-2010 | 0.828*** | 0.808* | 0.673 | 0.400*** | 0.738 |
|  | [0.758,0.905] | [0.659,0.990] | [0.424,1.069] | [0.248,0.645] | [0.505,1.080] |
| *Graduation year X GPA* |  |  |  |  |  |
| 1998-2004 X Low GPA | 1.147* | 1.176 | 0.773 | 1.745 | 1.084 |
|  | [1.004,1.310] | [0.872,1.588] | [0.386,1.548] | [0.943,3.229] | [0.680,1.728] |
| 2005-2010 X Low GPA | 1.354*** | 1.040 | 0.841 | 2.004* | 0.780 |
|  | [1.180,1.554] | [0.765,1.414] | [0.448,1.577] | [1.014,3.960] | [0.459,1.327] |
| N (individuals) | 1737374 | 254574 | 85546 | 73569 | 101589 |
| N (observations) | 13765754 | 1999328 | 672691 | 561081 | 792174 |

Table reports hazard ratios, with 95% confidence intervals in brackets. * p<0.05, ** p<0.01, *** p<0.001. Abbreviations: ref = reference category; GPA = Grade point average. Western background = at least one parent born in Europe or North America and not parent born outside Europe or North America. Non-Western background = at least one parent born outside Europe or North America. Estimates based on logistic regression models.

**Table H2. Cause-specific discrete time proportional hazard models with deaths of despair vs. all other causes of death as outcomes.**

|  | *Native-born & Swedish background* | *Native-born & Western background* | *Native-born & non-Western background* | *Foreign-born & Western background* | *Foreign-born & non-Western background* |
| --- | --- | --- | --- | --- | --- |
| *GPA (ref: medium/high GPA)* |  |  |  |  |  |
| Low GPA | 3.074*** | 2.913*** | 3.798** | 1.421 | 2.865*** |
|  | [2.603,3.630] | [2.122,4.000] | [1.613,8.943] | [0.615,3.282] | [1.640,5.005] |
| *Graduation year (ref: 1990-1997)* |  |  |  |  |  |
| 1998-2004 | 1.275** | 0.949 | 0.696 | 0.774 | 1.007 |
|  | [1.097,1.482] | [0.678,1.328] | [0.309,1.568] | [0.369,1.624] | [0.558,1.820] |
| 2005-2010 | 1.304*** | 1.215 | 0.894 | 1.066 | 1.043 |
|  | [1.123,1.515] | [0.886,1.666] | [0.442,1.810] | [0.520,2.183] | [0.544,1.999] |
| *Graduation year X GPA* |  |  |  |  |  |
| 1998-2004 X Low GPA | 1.118 | 1.058 | 1.232 | 1.488 | 0.761 |
|  | [0.893,1.401] | [0.662,1.691] | [0.414,3.670] | [0.486,4.552] | [0.348,1.662] |
| 2005-2010 X Low GPA | 1.422** | 0.972 | 0.729 | 1.603 | 0.426 |
|  | [1.142,1.772] | [0.618,1.529] | [0.272,1.957] | [0.541,4.755] | [0.174,1.047] |
| N (individuals) | 1737374 | 254574 | 85546 | 73569 | 101589 |
| N (observations) | 13765754 | 1999328 | 672691 | 561081 | 792174 |

Table reports hazard ratios, with 95% confidence intervals in brackets. * p<0.05, ** p<0.01, *** p<0.001. Abbreviations: ref = reference category; GPA = Grade point average. Western = at least one parent born in Europe or North America, non-Western background = at least one parent born outside Europe or North America. Estimates based on logistic regression models. Only results for deaths of despair shown in the table

**Table H3.** **Country or region of birth of the participants and the study participants’ parents**

| **Participant’s country of birth** |  |  |
| --- | --- | --- |
| *Country or region* | *N* | *% of sample* |
| Africa | 13,513 | 0.60 |
| Asia | 70,200 | 3.12 |
| EU28 except Nordic | 21,432 | 0.95 |
| Europe except EU28 and Nordic | 36,618 | 1.63 |
| North America | 3,906 | 0.17 |
| Nordic except Sverige | 11,623 | 0.52 |
| Oceania | 368 | 0.02 |
| South America | 16,524 | 0.73 |
| Soviet Union | 991 | 0.04 |
| Sweden | 2,077,756 | 92.22 |
| Not known or stateless | 15 | 0.00 |
| **Mother’s country of birth** |  |  |
| *Country or region* | *N* | *% of sample* |
| Africa | 20,132 | 0.90 |
| Asia | 88,528 | 3.94 |
| EU28 except Nordic | 54,643 | 2.43 |
| Europe except EU28 and Nordic | 70,576 | 3.14 |
| North America | 5,747 | 0.26 |
| Nordic except Sverige | 104,062 | 4.63 |
| Oceania | 511 | 0.02 |
| South America | 19,923 | 0.89 |
| Soviet Union | 2,272 | 0.10 |
| Sweden | 1,879,698 | 83.69 |
| Not known or stateless | 19 | 0.00 |
| **Father’s country of birth** |  |  |
| *Country or region* | *N* | *% of sample* |
| Africa | 24,515 | 1.11 |
| Asia | 84,036 | 3.80 |
| EU28 except Nordic | 59,848 | 2.70 |
| Europe except EU28 and Nordic | 73,017 | 3.30 |
| North America | 6,684 | 0.30 |
| Nordic except Sverige | 84,514 | 3.82 |
| Oceania | 620 | 0.03 |
| South America | 19,439 | 0.88 |
| Soviet Union | 1,040 | 0.05 |
| Sweden | 1,858,799 | 84.01 |
| Not known or stateless | 24 | 0.00 |

**Table H4. Most common country of birth apart from Sweden in ages 15-24, 2000-2018.**

| *Country* | *Share of population aged 15-24* |
| --- | --- |
| Iraq | 1.67% |
| Former Yugoslavia, including Bosnia | 1.56% |
| Somalia | 0.8% |
| Syria | 0.7% |
| Afghanistan | 0.61% |
| Iran | 0.56% |
| Poland | 0.48% |
| Thailand | 0.36% |
| Turkey | 0.32% |
| Finland | 0.31% |

Source: Statistics Sweden (2024)

**References**

Allebeck, P., Allgulander, C., Henningsohn, L., & Jakobsson, S. W. (1991). Causes of death in a cohort of 50 465 young men – Validity of recorded suicide as underlying cause of death. *Scandinavian Journal of Social Medicine*, *19*(4), 242-247. https://doi.org/10.1177/140349489101900405

Austin, P. C., & Fine, J. P. (2017). Practical recommendations for reporting Fine-Gray model analyses for competing risk data. *Statistics in Medicine*, *36*(27), 4391-4400. https://doi.org/https://doi.org/10.1002/sim.7501

Fine, J. P., & Gray, R. J. (1999). A Proportional Hazards Model for the Subdistribution of a Competing Risk. *Journal of the American Statistical Association*, *94*(446), 496-509. https://doi.org/10.2307/2670170

Gunnell, D., Bennewith, O., Simkin, S., Cooper, J., Klineberg, E., Rodway, C., Sutton, L., Steeg, S., Wells, C., Hawton, K., & Kapur, N. (2013). Time trends in coroners' use of different verdicts for possible suicides and their impact on officially reported incidence of suicide in England: 1990–2005. *Psychological Medicine*, *43*(7), 1415-1422. https://doi.org/10.1017/S0033291712002401

Lau, B., Cole, S. R., & Gange, S. J. (2009). Competing Risk Regression Models for Epidemiologic Data. *American Journal of Epidemiology*, *170*(2), 244-256. https://doi.org/10.1093/aje/kwp107

Schmid, M., & Berger, M. (2021). Competing risks analysis for discrete time-to-event data. *WIREs Computational Statistics*, *13*(5), e1529. https://doi.org/https://doi.org/10.1002/wics.1529

Spark, T. L., Adams, R. S., Hoffmire, C. A., Forster, J. E., & Brenner, L. A. (2022). Are We Undercounting the True Burden of Mortality Related to Suicide, Alcohol Use, or Drug Use? An Analysis Using Death Certificate Data From Colorado Veterans. *American Journal of Epidemiology*, *192*(5), 720-731. <https://doi.org/10.1093/aje/kwac194>

Statistics Sweden (2024). Befolkning efter födelseland, år och ålder. Retrieved from <https://www.statistikdatabasen.scb.se/pxweb/sv/ssd/START__BE__BE0101__BE0101E/FodelselandArK/> Accessed 2024.11.25

Tilstra, A. M. (2023). Invited Commentary: Stop Analyzing Suicides, Drug-Related Deaths, and Alcohol-Related Deaths Together. *American Journal of Epidemiology*, *192*(5), 732-733. https://doi.org/10.1093/aje/kwad002

VanderWeele, T. J., & Knol, M. J. (2014). A Tutorial on Interaction. *Epidemiologic Methods*, *3*(1), 33-72. https://doi.org/doi:10.1515/em-2013-0005
